# Integrated bioinformatics and single-cell transcriptomics identify mitochondrial dysfunction-driven biomarkers for preeclampsia diagnosis

**DOI:** 10.3389/fmolb.2026.1922190

**Published:** 2026-09-23

**Authors:** Lin Lin, Xiaowu Wang, Chengxiu Zhu, Kejun Ye, Dan Zhang, Ruohua Li, Mengjia Peng

**Affiliations:** 1 Department of Gynecology and Obstetrics, The Third Affiliated Hospital of Wenzhou Medical University, Rui’an, China; 2 Department of Burn and Skin Repair Surgery, The Third Affiliated Hospital of Wenzhou Medical University, Rui’an, China

**Keywords:** bioinformatics, diagnostic biomarkers, mitochondrial dysfunction, preeclampsia, single-cell analysis

## Abstract

**Background:**

Preeclampsia is a complex pregnancy disorder characterized by hypertension and proteinuria that pose significant risks to maternal and fetal health. Current diagnostic and treatment methods are limited, necessitating the identification of effective biomarkers and therapeutic targets.

**Methods:**

This study utilized bioinformatics methods, including differential expression analysis, weighted gene co-expression network analysis, protein–protein interaction networks, and machine learning algorithms, to identify critical mitochondrial genes associated with preeclampsia. Enrichment analysis was performed using gene set enrichment analysis, gene set variation analysis, gene ontology, and the Kyoto Encyclopedia of Genes and Genomes. Receiver operating characteristic analysis and SHapley Additive exPlanations analysis were used to evaluate the diagnostic performance of key genes and diagnostic models. Single-cell analysis provided insights into cellular heterogeneity and interactions. The identified key genes were validated via RT-qPCR.

**Results:**

Six mitochondrial key genes (*SOD1*, *ACSF2*, *ABAT*, *ACSS1*, *ALDH4A1*, *PDHA1*) and diagnostic models were identified, demonstrating robust diagnostic accuracy. Functional enrichment implicated these genes in oxidative phosphorylation, TCA cycle, and fatty acid metabolism. Single-cell analysis revealed trophoblast subpopulations as key sites of mitochondrial dysregulation. RT-qPCR confirmed significant downregulation of *ABAT*, *PDHA1*, and *SOD1* in PE placentas.

**Conclusion:**

Genes and pathways related to preeclampsia identified based on bioinformatic analyses may have diagnostic and therapeutic utility. Future research should confirm their clinical potential in larger groups of individuals.

## 1 Introduction

Preeclampsia (PE) is a serious complication exclusive to pregnancy, primarily identified by the presence of high blood pressure and proteins in the urine, manifesting beyond the 20-week gestation mark (Author Anonymous, 2013). This condition affects approximately 2%–8% of pregnancies globally and is one of the foremost contributors to both maternal and perinatal mortality rates (Steegers et al., 2010). In the case of PE, there is potential for severe complications such as eclampsia and HELLP syndrome (hemolysis, elevated liver enzymes, and low platelet syndrome), which can pose a significant risk to the lives of both mother and fetus (Dimitriadis et al., 2023). The etiology of PE is multifactorial, involving a combination of genetic predispositions, environmental influences, infections, and abnormalities in trophoblast function. Additionally, maternal factors such as pre-existing hypertension, diabetes, obesity, and autoimmune disorders can increase the risk of developing PE (Bhattacharya et al., 2012). Currently, the diagnosis of PE relies primarily on clinical symptoms, while treatment focuses on lowering blood pressure, administering magnesium spasmolytics, and, if necessary, terminating the pregnancy (Wu et al., 2023). However, these diagnostic and treatment methods have notable drawbacks, including delayed detection, potential drug side effects, symptom relief without addressing the underlying condition, insufficient personalized treatment, and a heightened risk of preterm birth. Therefore, although current treatments have improved PE prognosis, there is an immediate need for more effective diagnostic markers and therapeutic targets.

Mitochondria are crucial for energy production, cell signaling, cell cycle regulation, and apoptosis (Picard et al., 2016). Appropriate trophoblast function is essential for placental health and fetal development (Brosens et al., 2011). However, mitochondrial dysfunction, including DNA damage, oxidative stress, and abnormal apoptotic pathways, plays a pivotal role in PE pathogenesis (Anton et al., 2019). This dysfunction contributes to trophoblast injury, placental abnormalities, endothelial damage, and systemic manifestations of PE, highlighting mitochondrial dysregulation as a critical factor in this disease (Phipps et al., 2019).

Despite extensive clinical investigations into PE, the precise genetic determinants governing its onset and pathogenesis remain incompletely characterized. To address this knowledge gap, our study implemented an integrative bioinformatics pipeline incorporating weighted gene co-expression network analysis (WGCNA), machine learning algorithms, and single-cell transcriptomic profiling to systematically identify mitochondrial-associated hub genes implicated in PE pathophysiology. Functional enrichment analyses revealed these genes’ critical involvement in oxidative phosphorylation, TCA cycle regulation, and fatty acid metabolism, with concomitant dysregulation of placental microenvironment alterations. Receiver operating characteristic (ROC) curve analysis, demonstrating robust predictive accuracy for key mitochondrial genes. A Random Forest model integrating all six hub genes achieved superior performance, outperforming traditional clinical markers. SHapley Additive exPlanations (SHAP) analysis further quantified feature contributions. Single-cell resolution analysis further delineated placental cellular heterogeneity, identifying trophoblast subpopulations as primary sites of mitochondrial dysfunction. Experimental validation via RT-qPCR confirmed significant downregulation of core mitochondrial genes (*ABAT*, *PDHA1*, *SOD1*) in PE placental tissues. This multimodal approach—synthesizing computational predictions with molecular validation—establishes a framework for identifying high-fidelity diagnostic biomarkers and mechanistically informed therapeutic targets, thereby advancing precision medicine strategies for PE management.

## 2 Materials and methods

### 2.1 Datasets download and processing

We downloaded the PE datasets GSE24129 (Nishizawa et al., 2011), GSE75010 (Leavey et al., 2016; Leavey et al., 2017; Christians et al., 2017; Leavey et al., 2018; Benton et al., 2018; Gibbs et al., 2019), GSE186257 (Horii et al., 2023), and GSE173193 (Zhang et al., 2022; Yang et al., 2021) from the Gene Expression Omnibus (GEO) database using the R package “GEOquery” (Barrett et al., 2013) (https://www.ncbi.nlm.nih.gov/geo/). The samples in GSE75010 and GSE24129 were all from *Homo sapiens*, with the placenta as the tissue source and GPL6244 as the chip platform. The GSE186257 dataset comprised samples from *H. sapiens*, with the placenta as the tissue source and GPL24676 as the sequencing platform. Dataset GSE75010 included 80 PE and 77 control samples, dataset GSE24129 included 8 PE and 8 control samples, whereas dataset GSE186257 was included in this study as a validation dataset, containing 26 PE samples and 18 control samples. Additionally, the dataset GSE173193, as a single-cell RNA sequencing (scRNA-seq) data, consisted of *H. sapiens* samples with placenta as tissue source, including 2 PE samples and 2 control samples. For specific information on sample sizes and other details, please refer to Table 1. After batch removal, datasets GSE24129 and GSE75010 were pooled and included in this study.

**TABLE 1:** GEO dataset information.

Series	Platform	Species	Tissue	PE samples	Control samples	
GSE75010	GPL6244	*Homo sapiens*	Placenta	80	77	Training
GSE24129	GPL6244	*Homo sapiens*	Placenta	8	8	Training
GSE186257	GPL24676	*Homo sapiens*	Placenta	26	18	Validation
GSE173193	GPL24676	*Homo sapiens*	Placenta	2	2	scRNA-seq

The MitoCarta3.0 database (Rath et al., 2021) (https://www.broadinstitute.org/mitocarta) offers extensive data regarding 1,136 genes associated with human mitochondria, including their submitochondrial positioning and associated functions. It also includes 149 mitochondria-related pathways. Additionally, it provides a comprehensive score for approximately 20,000 human genes based on mitochondrial localization. We utilized this database to identify 1,136 mitochondria-related genes (MitoRGs) and 149 mitochondria-related pathways (mitopathways) for this study. For further details, please refer to Supplementary Table S1.

The R package “sva” (Leek et al., 2012) was used to process the GSE24129 and GSE75010 datasets to generate a combined dataset consisting of 88 PE samples and 85 control samples. Subsequently, the combined dataset underwent probe annotation and standardization using the R package “limma” (Ritchie et al., 2015). Finally, principal component analysis (PCA) (Ben Salem and Ben Abdelaziz, 2021) was used to evaluate the batch processing effect of the combined dataset.

### 2.2 WGCNA and differentially expressed analysis

WGCNA is a robust method used for identifying co-expressed gene modules while simultaneously investigating their associations with gene networks, relevant phenotypes, and key genes. The R package “WGCNA” (Langfelder and Horvath, 2008) was used for the analysis. Initially, we focused on identifying the core modules within the combined dataset. To achieve this, we used the Hclust function to detect and exclude any outlier samples. A clustering dendrogram, based on gene correlation coefficients, was created to represent distinct gene modules through its branches. We identified a soft-thresholding power between 1 and 20, ensuring R^2^ > 0.9. Specifically, we used the “pickSoftThreshold” function to calculate the scale-free topology fit index for various powers and selected the lowest power that resulted in an R^2^ value greater than 0.9. After determining the optimal power, the adjacency matrix was converted into a topological overlap matrix (TOM). Subsequently, 1-TOM was computed to quantify the interconnectedness of the genes, and gene modules were identified based on the TOM difference measure, using the average linkage hierarchical clustering method. The minimum module size considered in this analysis was 50. Next, we assessed the correlation of each gene within the modules in relation to both the PE and control groups. We used the R package “limma” to identify differentially expressed genes (DEGs) in the combined dataset, with an adjusted p-value (*adj.p*) < 0.05 and | log2 fold change (logFC)| > 0.1. An *adj. p* < 0.05 with logFC >0.1 indicated upregulation of DEGs, whereas an *adj. p* < 0.05 with logFC < −0.1 indicated downregulation. We employed the R package “ggplot2” to generate a volcano plot to visualize the findings of the analysis. We intersected the most significant WGCNA module with DEGs and MitoRGs and visualized the results using a Venn diagram. We obtained a set of mitochondria-associated differentially expressed genes (MitoRDEGs) for inclusion in subsequent analyses.

### 2.3 Enrichment analysis

Gene set enrichment analysis (GSEA) (Hänzelmann et al., 2005a) is a bioinformatic technique that analyzes gene expression data to identify gene sets associated with specific biological functions or pathways. It also evaluates the enrichment of these gene sets and their statistical significance under different conditions. When conducting GSEA, we utilized the R package “ClusterProfiler” to analyze the data sets from “c2. cp.all.v2022.1. Hs. symbols.gmt [All Canonical Pathways](3050)” downloaded from the MSigDB database (Liberzon et al., 2011) (https://www.gsea-msigdb.org/). The parameters included 1000 computations, a minimum of 10 genes, a maximum of 500 genes per set, and p-value correction via the Benjamini–Hochberg (BH) procedure. The criteria for significant enrichment were set as false discovery rate (FDR) < 0.25 and *adj. p* < 0.05.

Gene set variation analysis (GSVA) (Hänzelmann et al., 2005b) uses gene expression data to evaluate gene set activity in individual samples. This analysis helps to reveal variations in gene set activity across samples and their significance in biology or disease. We applied the R package “GSVA” to the combined dataset using the mitopathway gene set from the MitoCarta3.0 database. The enrichment method was applied, and the probability distribution was Gaussian. We obtained differential expression pathways using the R package “limma”. Selection criteria were set to *adj. p* < 0.05 and |logFC| > 0.25. The R packages “heatmap” and “ggplot2” were used to produce bar charts and heat maps to enhance visualization in the context of academic research.

Gene ontology (GO) analysis (Mi et al., 2019) is a bioinformatics method used to explain the roles of gene functions and gene products in biological processes (BP), cellular components (CC), and molecular functions (MF). The Kyoto Encyclopedia of Genes and Genomes (KEGG) (Yu et al., 2012) database (27) is an extensive resource for investigating the functionality and practical applications of biological systems. The MitoRDEGs were subjected to GO and KEGG enrichment analyses using the R package “clusterProfiler”. Genes were included in the analysis if both adj. p and FDR were <0.05, indicating statistical significance. We used the BH procedure for p-value adjustment and generated a lollipop chart for visualization.

### 2.4 Protein–protein interaction (PPI) network construction and key genes screening

The PPI network consists of interacting proteins. We explored the relationships between known and predicted proteins using the Search Tool for Interacting Genes/Proteins (STRING) database (Yu et al., 2019) (https://cn.string-db.org/). We developed a PPI network for mitochondria using the STRING database. The minimum interaction score was set to 0.400. STRING results were visualized using Cytoscape (version 3.10.2) (Shannon et al., 2003). Additional analysis was conducted with the cytoHubba (Chin et al., 2014) plug-in using the Degree algorithm. We identified 15 genes with the highest degree as key genes for further analysis and used them to construct subnetworks.

### 2.5 Identifying key feature genes using three machine learning programs

To identify key feature genes associated with PE, we employed three machine learning algorithms: Least Absolute Shrinkage and Selection Operator (LASSO), Support Vector Machine-Recursive Feature Elimination (SVM-RFE), and Random Forest. The LASSO method is a statistical approach that minimizes the loss function with an added penalty term proportional to the absolute value of the feature coefficient (L1 regularization). This effectively reduces some coefficients to zero, thereby facilitating feature selection. LASSO is particularly useful for addressing classification problems characterized by high-dimensional features, as it can handle multicollinearity and select a sparse set of features. We employed the “glmnet” (Engebretsen and Bohlin, 2019) package in R to perform LASSO regression with ten-fold cross-validation. Ten-fold cross-validation was chosen to ensure robust model performance and prevent overfitting by dividing the data into ten subsets, training the model on nine subsets, and validating it on the remaining subset. SVM-RFE aims to select the most important features by recursively building a model and removing the least-weighted features. The weightings of each feature are calculated separately in each iteration, and then the least-weighted feature is removed until a predetermined number of features is reached or some stopping criterion is met. SVM-RFE was implemented using the “e1071” (Wilkerson et al., 2014) package in R with ten-fold cross-validation. This method was chosen for its ability to handle non-linear relationships and its effectiveness in selecting a subset of features that contribute most to the classification task. Random forest is an ensemble learning algorithm that identifies and selects the most important features by building multiple decision trees and evaluating the importance of each feature in the tree. We implemented random forest using the “randomForest” (Izmirlian, 2004; Franz et al., 2018) package in R with ten-fold cross-validation. Random Forest was selected for its robustness to overfitting, ability to handle large datasets with higher dimensionality, and its capability to provide feature importance scores, which are useful for identifying key genes. Venn diagrams were constructed to visualize the intersections among feature genes identified by the three machine-learning methods. Only genes independently selected by LASSO, SVM-RFE, and random forest were retained as the final consensus feature genes. The GeneMANIA database (Franz et al., 2018) (https://genemania.org) is an online tool for predicting functional associations between genes that helps researchers explore gene functions and interrelationships by integrating multiple data sources. We utilized GeneMANIA to create a co-expression network involving key genes.

### 2.6 Analysis of key genes expression differences and correlations

A heatmap of key genes from the combined dataset was generated using the R package “ComplexHeatmap”. We utilized the R package “ggplot2” to generate group comparison plots, taking into account the expression levels of key genes. Subsequently, we utilized the R package “RCircos” for chromosomal mapping of key genes. We conducted pairwise correlation analysis of the key genes using the Spearman statistical method. We then created a correlation heatmap with the R package “corrplot” and a chord diagram using the R packages “igraph” and “ggraph”. Finally, we used the R package “GOSemSim” to conduct Friends analysis of the key genes.

### 2.7 ROC analysis and construction and interpretation of the predictive model

We first utilized the R package “pROC” (Robin et al., 2011) to generate ROC curves for key genes and calculated the area under the curve (AUC) values to evaluate their diagnostic efficiency (0.7–0.9 indicating moderate accuracy, >0.9 high accuracy), with validation performed using the GSE186257 dataset. Subsequently, we developed a predictive model incorporating ten machine learning algorithms to assess the impact of each key gene on PE diagnosis. ROC curves and AUC values were employed to evaluate the diagnostic performance of all models, prioritizing the model with the highest AUC. Decision curve analysis (DCA) and calibration curves were applied to validate the model’s clinical utility and robustness. To enhance interpretability, SHAP (Aas et al., 2021) analysis was implemented using the SHAP method in R, where positive/negative SHAP values reflected directional effects on risk prediction (increase/decrease). The “shapviz” package generated global feature importance rankings through mean absolute SHAP values, visualized via bar plots (overall importance) and beeswarm plots (feature value-SHAP value distributions). Waterfall plots were created for representative cases to provide local interpretability of model decisions. This comprehensive SHAP-based framework quantitatively delineated the contributions of key feature genes to PE risk prediction, establishing a transparent and clinically actionable diagnostic model.

### 2.8 Single-cell analysis involves quality control, clustering, and annotation

We used the “Seurat” package (v5.1.0) (Stuart et al., 2019) for quality control, dimensionality reduction, clustering, and cell annotation. Initially, raw data from the GSE173193 dataset were imported, and Seurat objects were created for each sample with at least 5 cells and 300 features. These objects were then merged for analysis. Subsequently, we calculated mitochondrial and hemoglobin gene expression percentages, filtering out cells with more than 25% mitochondrial and less than 1% hemoglobin content. The data were then normalized, and highly variable genes were identified. Following this, we scaled the data, performed PCA, and used the Harmony algorithm (Korsunsky et al., 2019) for batch effect correction. For visualization and clustering, Uniform Manifold Approximation and Projection (UMAP) was employed using the Louvain algorithm at a resolution of 0.3. Finally, cell types were identified using known lineage markers (Gong et al., 2024) and the CellMarker website (http://xteam.xbio.top/CellMarker/), which provides a mapping of human placental cells.

### 2.9 Milo differential abundance analysis

We used the “miloR” R package (Dann et al., 2022) for Milo differential abundance analysis to examine changes in cell neighborhood composition across conditions. Starting with a Seurat object, we created a SingleCellExperiment object and initialized a Milo object. A k-nearest neighbor graph was built with k = 30 and d = 50. Neighborhoods were defined using the makeNhoods function with a 0.2 random sampling of graph vertices. Cell counts in neighborhoods were calculated with the countCells function, identifying samples by their original IDs. Differential abundance testing was conducted using the testNhoods function with a design matrix including sample IDs and group info. Results were visualized on a neighborhood graph with nodes colored by LogFC, and as beeswarm plots grouped by cell type to show logFC.

### 2.10 AUCell score, difference analysis and enrichment analysis

To evaluate mitochondrial gene activity across cell subsets, we used the “AUCell” R package (Aibar et al., 2017) to calculate the AUCell score of MitoRGs for each cell subset, setting a maximum rank at 5% of total cells. For key subsets, we calculated and log-transformed average gene expression levels. The FindMarkers function was employed to identify differentially expressed genes between PE and control groups, with thresholds of 0.25 for minimum percentage and 0 for logFC. Additionally, ggplot2 was used to visualize the proportion and number of key genes, as well as the top 5 up- and downregulated genes and cell subsets. Finally, we explored the functional enrichment of differentially expressed genes in key subsets using GO and KEGG pathway analysis with the “clusterProfiler” package in R.

### 2.11 Cell trajectory inference

Initially, we used the “CytoTRACE2” R package (Gulati et al., 2020) to calculate CytoTRACE2 scores. CytoTRACE2 is a computational tool designed to predict cell differentiation states and trajectories from single-cell RNA sequencing data, enhancing the understanding of cellular development and heterogeneity. Subsequently, for cell differentiation trajectories, we conducted an unsupervised pseudo-time analysis with “Monocle 3” R package (Cao et al., 2019). The Monocle 3 object was processed with PCA, and UMAP was employed for dimensionality reduction to visualize the data in a lower-dimensional space. The learn_graph function was then utilized to create a principal graph for the trajectory, and cells were ordered using the order_cells function. Trajectory roots were chosen based on the pseudotemporal gradient and CytoTRACE score. Finally, we utilized the R package “destiny” for diffusion map analysis (Haghverdi et al., 2015), a technique that models data geometry through diffusion processes. By mapping the 3D diffusion map, we can show the cell distribution of the first three components (DC1, DC2, DC3), revealing potential cell development pathways. The scRNA-seq gene expression matrix was converted into an ExpressionSet object, and the diffusion map was computed with the DiffusionMap function. The diffusion map were visualized in 3D using the “plot3d” package. The analysis incorporated CytoTRACE2 rankings, based on gene expression per cell, to clarify cell differentiation states.

### 2.12 Cell-cell interaction analysis

Cell-cell interaction analysis was conducted using the “CellChat” R package (Jin et al., 2021) (version 2.1.2), leveraging known ligand-receptor interactions. A CellChat object was created from single-cell RNA sequencing data, utilizing the human CellChat database to focus on secreted signaling interactions. The data was processed to identify highexpressed genes and ligand-receptor pairs, which were mapped onto the human protein-protein interaction network. Communication probabilities were calculated to infer the interaction network, excluding interactions with fewer than three cells.

### 2.13 Patient and tissue specimens

Placental specimens were collected from 20 expectant mothers who underwent cesarean delivery at the Third Affiliated Hospital of Wenzhou Medical University. Ten mothers presented with PE, while ten served as healthy controls matched for gestational age. Ethical approval was granted by the Research Ethics Committee of the Ruian People’s Hospital (approval number: YJ2024120). Written consent was obtained from all participants. The inclusion criteria for the PE group were as follows: Women between the ages of 20 and 40 years who had a protein volume of 0.3 g or more in their urine 24 h after 20 weeks of gestation and a blood pressure of 140/90 mm Hg or higher, with no other obvious pregnancy-related or other complications. After delivery, we extracted a tissue sample from the central region of the placenta and preserved it at −80 °C for long-term storage.

### 2.14 RT-qPCR (quantitative reverse transcription polymerase chain reaction)

Total RNA was extracted using a Tissue Total RNA Isolation Kit V2 (Vazyme) and converted into cDNA using HiScript III All-in-one RT SuperMix (Vazyme). Subsequently, RT-qPCR was conducted using Taq Pro Universal SYBR qPCR Master Mix (Vazyme) on a CFX Connect Real-Time PCR System (Bio-Rad, Hercules, CA, United States of America). The 2^−ΔΔCT^ method was employed to quantify relative gene expression, using *GAPDH* as the reference gene.

### 2.15 Statistical analysis

Data processing and analysis were conducted using R software (Version 4.3.3). We used the independent Student’s *t*-test to assess the statistical significance of normally distributed variables when comparing two continuous groups, unless otherwise specified. We employed the Mann–Whitney U-Test (Wilcoxon rank-sum test) to examine differences in variables that did not follow a normal distribution. The Kruskal–Wallis test was used for comparisons involving three or more groups. Spearman’s correlation analysis was used to compute the correlation coefficients among various molecules. All p-values are reported bilaterally, with p < 0.05 indicating statistical significance.

## 3 Results

### 3.1 Technical roadmap

The technical roadmap of this study is shown in Figure 1.

**FIGURE 1:**
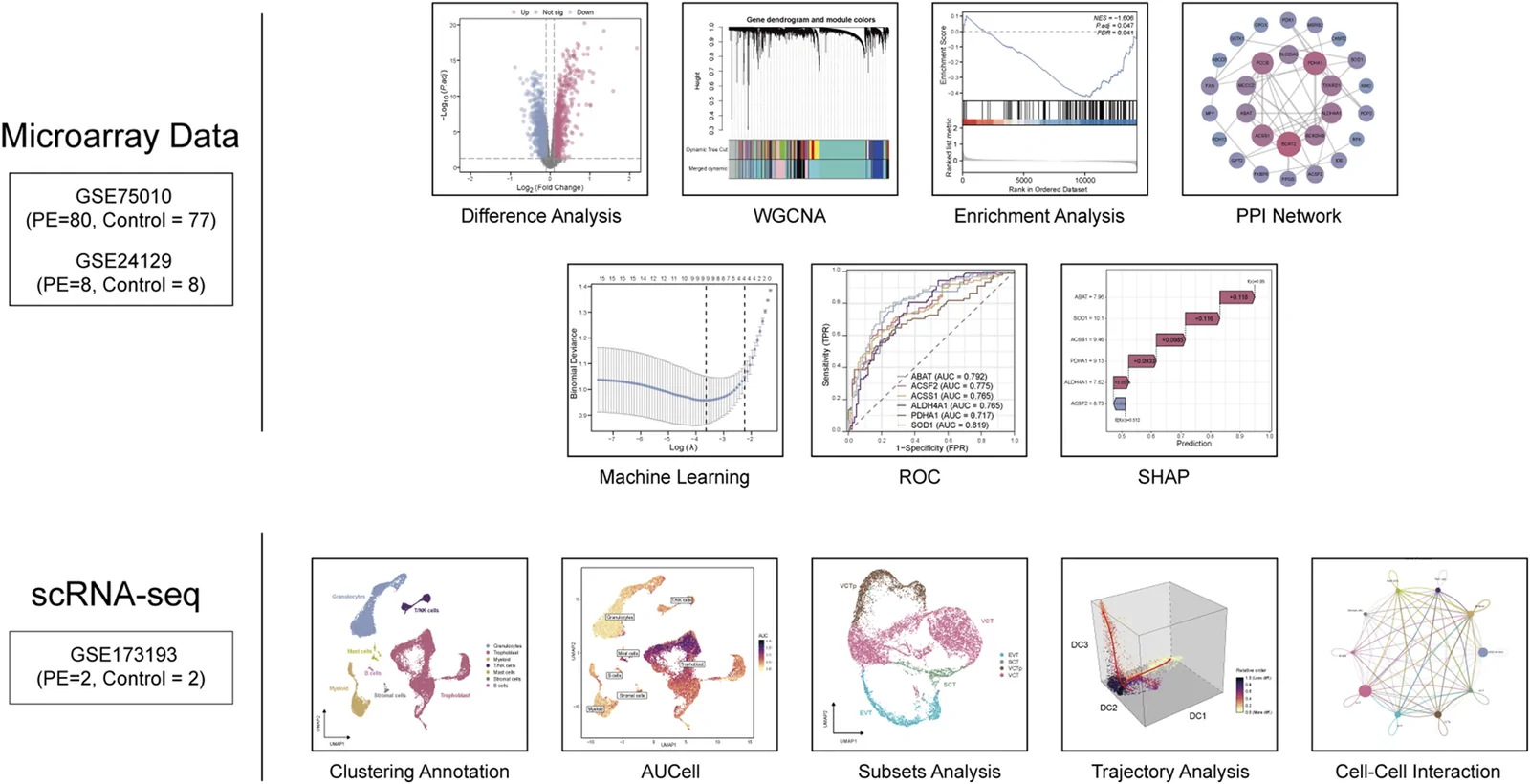
Technical roadmap.

### 3.2 Merging of PE datasets

The R “sva” package was used to eliminate batch effects from the PE datasets GSE24129 and GSE75010, resulting in a combined dataset. Distribution box plots (Supplementary Figures S1A,B) were used to compare the dataset expression values before and after batch-effect removal. We employed PCA to examine the distribution of low-dimensional features in the datasets, both before and after the removal of batch effects (Supplementary Figures S1C,D). The distribution box plots and PCA plots indicated successful elimination of batch effects in the combined dataset.

### 3.3 WGCNA and differentially expressed analysis

We began the WGCNA of the combined dataset, using a soft-thresholding power of 7 (Figure 2A). Subsequently, modules and a hierarchical clustering tree of all genes were constructed based on a topological overlap matrix (Figure 2B). We identified 24 distinct co-expression modules (Figure 2C), which were subsequently clustered and merged when the dissimilarity was less than 0.5, resulting in a total of 10 modules (Figure 2D). Further analysis indicated that the blue module exhibited the strongest correlation with the groups (Figure 2E), with a correlation coefficient of 0.67 and p-value of 1e−23). Therefore, we chose the blue module for further analysis (Supplementary Table S2). Based on the criteria of |logFC| > 0.1 and *adj. p* < 0.05, a total of 2,462 DEGs were identified in the combined dataset through differential analysis (Supplementary Table S3), comprising 965 upregulated genes (logFC >0.1 and *adj. p* < 0.05) and 1387 downregulated genes (logFC < −0.1 and *adj. p* < 0.05), as illustrated in Figure 2F. The intersection of DEGs, MitoRGs, and WGCNA MEblue was represented as a Venn diagram, resulting in 43 MitoRDEGs (Figure 2G). Detailed results are presented in Supplementary Table S4.

**FIGURE 2:**
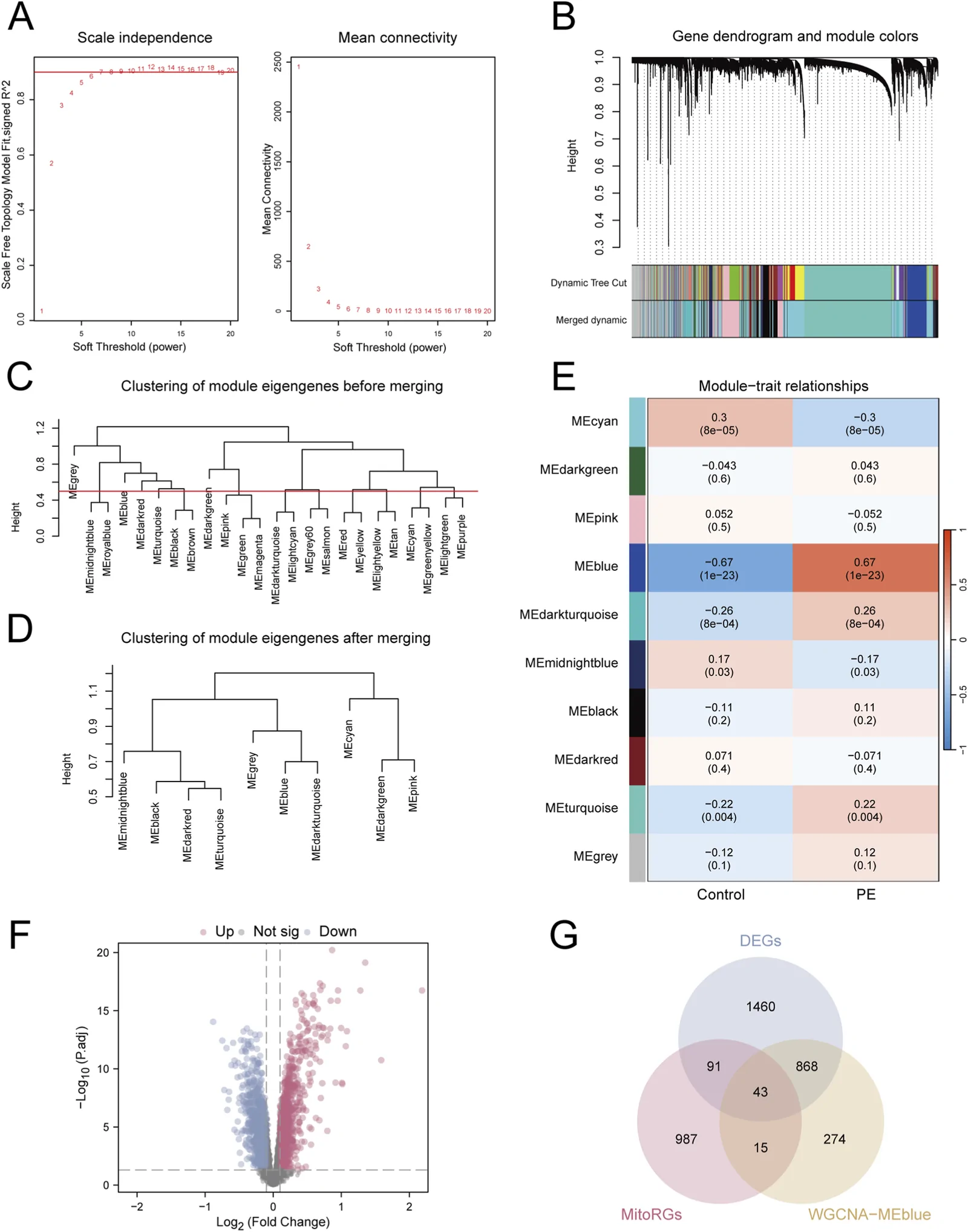
Identification of DEGs associated with MitoRGs using WGCNA. **(A)** The display of the scale-free network effectively illustrates the optimal soft threshold in WGCNA. **(B)** Results of the clustering analysis for genes, showing a hierarchical clustering dendrogram in the upper section and a representation of gene modules in the lower section. **(C)** Clustering of module eigengenes before merging. **(D)** Clustering of module eigengenes after merging. **(E)** Correlation analysis among the gene cluster modules. **(F)** Volcano plot illustrating the differential expression levels between the PE group and control group within the integrated dataset. **(G)** Venn diagram depicting the intersections among DEGs, MitoRGs, and the WGCNA module eigengene MEblue within the aggregated dataset. The screening criteria for DEGs were p. adj <0.05 and |logFC| > 0.1.

### 3.4 Enrichment analysis

Our GSEA results demonstrate the association between gene expression in the dataset and diverse biological processes, cellular components, and molecular functions, as depicted in Figure 3A. The combined dataset showed significant enrichment in pathways, including the citric acid (TCA) cycle and respiratory electron transport (Figure 3B), amino acid metabolism (Figure 3C), GABA (gamma-aminobutyric acid) receptor activation (Figure 3D), mitochondrial electron transport chain system (Figure 3E), mitochondrial complex I assembly model OXPHOS system (Figure 3F), DNA repair (Figure 3G), and other relevant biological functions and signaling pathways. Detailed results are presented in Supplementary Table S5.

**FIGURE 3:**
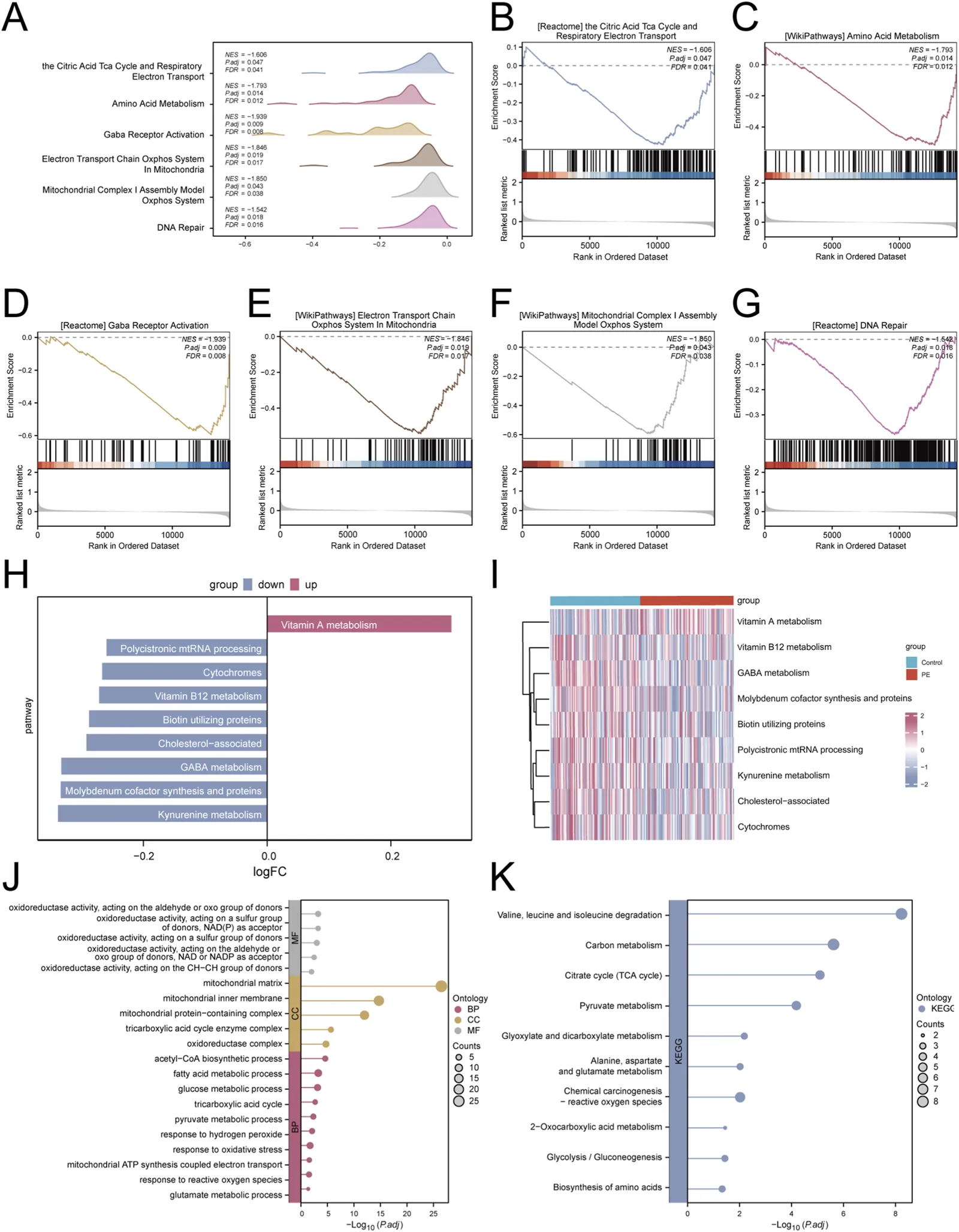
Enrichment analysis for combined dataset. **(A)** A total of six biological functions of GSEA in the combined dataset were illustrated using a mountain map. **(B–G)** GSEA revealed PE samples were significantly enriched in the citric acid TCA cycle and respiratory electron transport **(B)**, amino acid metabolism **(C)**, GABA receptor activation **(D)**, electron transport chain system in mitochondria **(E)**, mitochondrial complex I assembly model OXPHOS system **(F)**, and DNA repair **(G)**. **(H)** The bar plot depicts the top 9 differentially expressed pathways in the combined dataset using the mitopathways gene set. Pink indicates high expression in the PE group; blue indicates low expression in the PE group. The screening criteria were p. adj <0.05 and |logFC| > 0.25. **(I)** Heatmap illustrating the expression levels of the mitopathways in different groups. Pink indicates high expression; blue indicates low expression. **(J)** 43 lollipop charts showing GO analysis of MitoRDEGs. **(K)** 43 Lollipop charts showing KEGG analysis of MitoRDEGs.

Simultaneously, we conducted gene set variation analysis (GEVA) to explore the enrichment of the combined dataset in the mitopathway gene set. The PE group showed increased expression in vitamin A metabolism but decreased expression in kynurenine metabolism, molybdenum cofactor synthesis and proteins, GABA metabolism, cholesterol-associated, biotin-utilizing proteins, vitamin B12 metabolism, cytochromes, and polycistronic mtRNA processing. These results satisfied the criteria of |logFC| > 0.1 and *adj. p* < 0.05 (Figures 3H,I). Detailed results are presented in Supplementary Table S6.

GO and KEGG enrichment analysis revealed that the 43 MitoRDEGs are primarily associated with BP, such as acetyl coenzyme A (CoA) biosynthesis, fatty acid metabolism, glucose metabolism, and the tricarboxylic acid cycle. The CC includes the mitochondrial matrix and the inner mitochondrial membrane. MF has oxidoreductase activity, acting on the aldehyde or oxo groups of donors, and on the sulfur groups of donors with NAD(P) as an acceptor. The biological pathways included the citrate cycle (TCA cycle), pyruvate metabolism, aspartate and glutamate metabolism, glycolysis/gluconeogenesis, and amino acid biosynthesis (Figures 3J,K). Detailed results are presented in Supplementary Table S7.

### 3.5 Construction of PPI network and identification of top 15 key genes

Firstly, a PPI network comprising 43 MitoRDEGs was constructed using the STRING database. The PPI network results revealed a correlation among 28 MitoRDEGs (Figure 4A). Next, the top 15 key genes were selected to construct a subnetwork of MitoRDEGs based on the degree values of the nodes in the PPI network (Figure 4B). The key genes selected for further analysis were: *BCAT2, PDHA1, PCCB, ACSS1, TXNRD1, ALDH4A1, BCKDHB, MCCC2, SLC25A5, ABAT, ACSF2, SOD1, FXN, MSRB2,* and *PDP2*.

**FIGURE 4:**
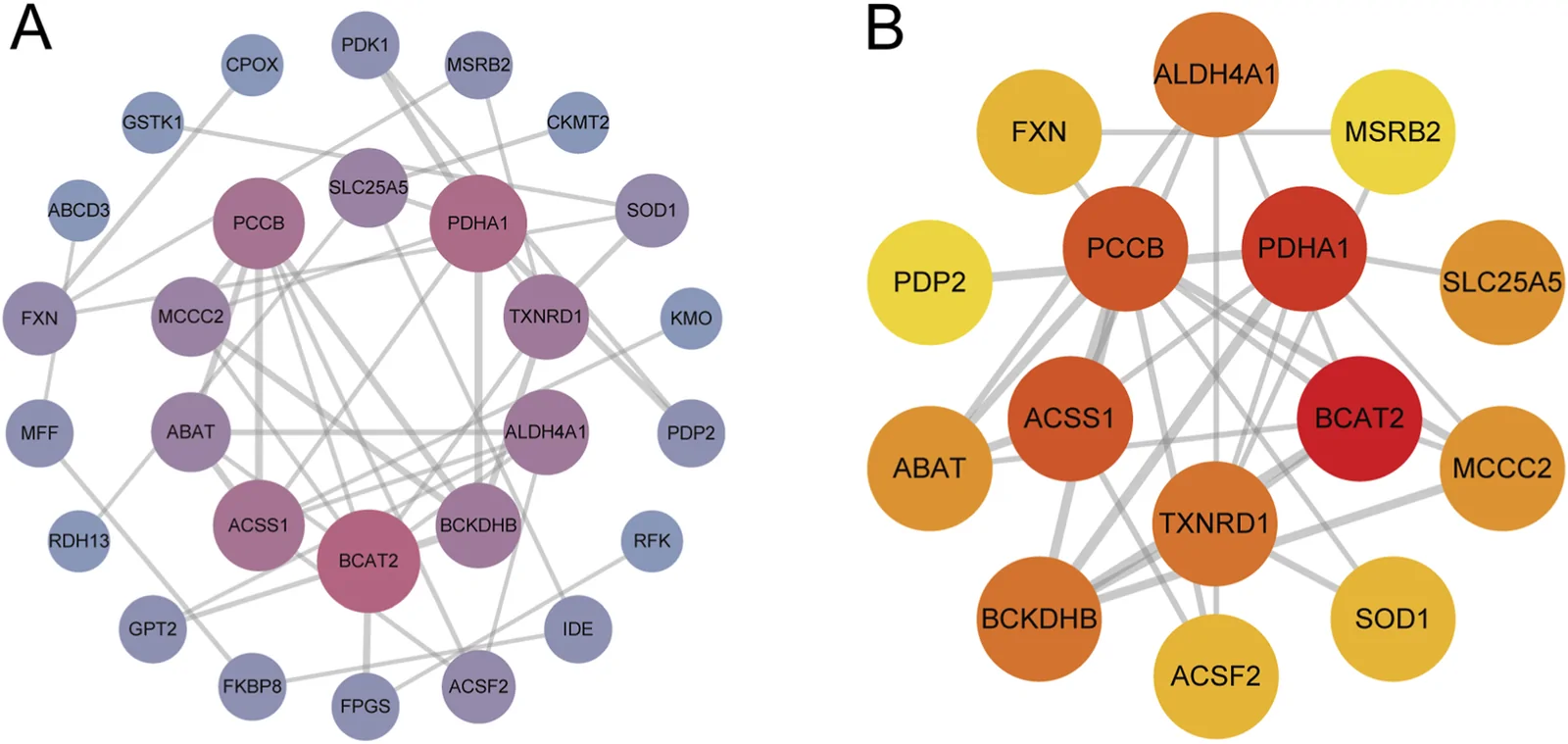
Analysis of the PPI network of MitoRDEGs identified the top 15 key genes. **(A)** PPI network. The size and color of nodes in the PPI network are positively correlated with their degree values. **(B)** The top 15 key genes within the PPI network were identified according to their degree values.

### 3.6 Utilizing LASSO, SVM-RFE, and random forest algorithms to identify key feature genes

We first identified nine key characteristic genes using the LASSO algorithm (Figures 5A,B). Subsequently, the SVM-RFE algorithm successfully identified seven genes (Figures 5C,D), followed by the random forest algorithm, which also identified eight genes (Figures 5E,F). Finally, we used a Venn diagram to compare the gene subsets identified by the three machine-learning algorithms. The intersection of the feature sets independently identified by LASSO, SVM-RFE, and random forest yielded six consensus genes: *superoxide dismutase 1* (*SOD1*), *acyl-CoA synthetase family member 2* (*ACSF2*), *4-aminobutyrate aminotransferase* (*ABAT*), *acyl-CoA synthetase short-chain family member 1* (*ACSS1*), *aldehyde dehydrogenase 4 family member A1* (*ALDH4A1*), and *pyruvate dehydrogenase E1 subunit alpha 1* (*PDHA1*) (Figure 5G). The complete screening sequence was MitoRGs–DEG–WGCNA intersection, PPI-based prioritization, and consensus selection using three machine-learning algorithms. The results are presented in the expanded Table 2, which summarizes the official gene names, principal mitochondrial functions, expression changes, and available evidence relevant to PE. We uploaded the six key genes to the GeneMANIA database to create a co-expression network. Different-colored lines represent the relationships between the genes, including predicted interactions, co-expression, shared protein domains, and genetic interactions (Figure 5H).

**FIGURE 5:**
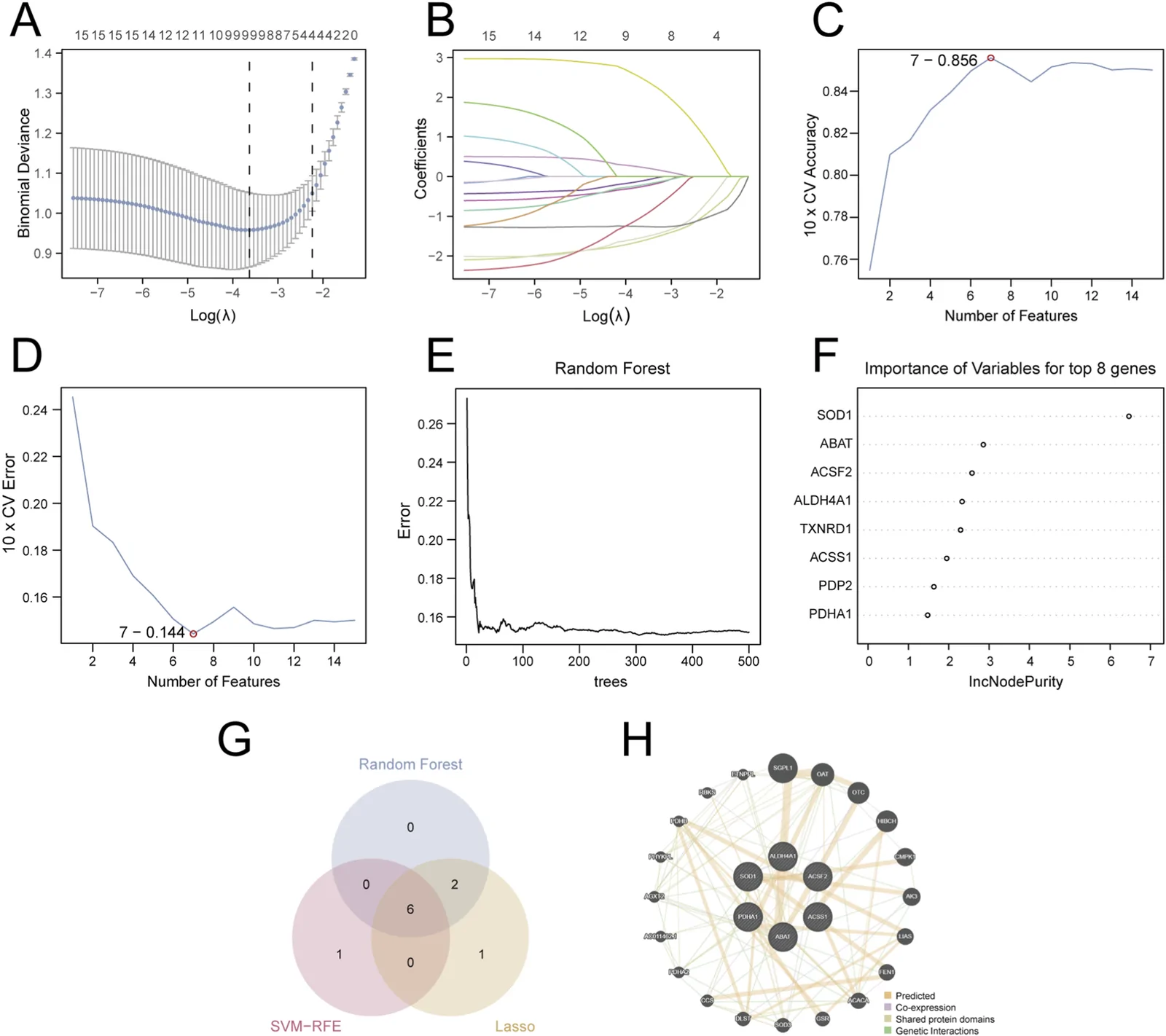
Screening key feature genes using machine learning algorithms. **(A,B)** Feature genes were identified using the LASSO algorithm. **(C,D)** The SVM-RFE algorithm was employed to identify and select significant feature genes. **(E,F)** Feature selection by random forest algorithm. **(G)** Venn diagram illustrating the overlapping key feature genes identified through LASSO, SVM-RFE, and random forest methods. **(H)** 6 key genes and their co-expression genes were analyzed via GeneMANIA.

**TABLE 2:** Full names, expression characteristics, biological functions, and evidence relevant to preeclampsia for the six key genes.

ID	Official full name	logFC	adj.P.Val	Principal mitochondrial function	Evidence relevant to preeclampsia
*ABAT*	4-Aminobutyrate aminotransferase	−0.322949662	1.53642E-10	Catalyzes GABA degradation to succinic semialdehyde and links GABA metabolism to the mitochondrial TCA cycle	Selected by the present data-driven pipeline and significantly downregulated in PE placentas
*ACSF2*	Acyl-CoA synthetase family member 2	0.193864187	7.50209E-09	Activates fatty acids to acyl-CoA thioesters and participates in mitochondrial lipid metabolism	Previously identified as an upregulated mitochondrial/programmed-cell-death-related biomarker in PE and validated by RT-qPCR, immunofluorescence, and Western blotting; its upregulation is consistent with the present bulk-transcriptomic result
*ACSS1*	Acyl-CoA synthetase short chain family member 1	−0.227989046	1.91618E-08	Generates mitochondrial acetyl-CoA from acetate, thereby supporting the TCA cycle and cellular energy metabolism	Selected by the present pipeline and downregulated in PE placentas
*ALDH4A1*	Aldehyde dehydrogenase 4 family member A1	−0.219853622	1.12458E-07	Catalyzes a step in mitochondrial proline degradation and contributes to glutamate production and redox metabolism	Selected by the present pipeline and downregulated in PE placentas. Its PE relevance is supported primarily by the current data and the broader evidence of mitochondrial gene dysregulation in hypertensive pregnancies
*PDHA1*	Pyruvate dehydrogenase E1 subunit alpha 1	−0.122150731	7.20868E-06	Catalytic subunit of the pyruvate dehydrogenase complex that converts pyruvate to acetyl-CoA, linking glycolysis to the mitochondrial TCA cycle	Selected by the present data-driven pipeline and significantly downregulated in PE placentas
*SOD1*	Superoxide dismutase 1	−0.426268896	1.38953E-12	Converts superoxide radicals to hydrogen peroxide and oxygen and contributes to cellular antioxidant defense	Reduced trophoblast SOD expression has been reported in PE, and a functional SOD1 promoter insertion/deletion polymorphism has been associated with PE susceptibility

### 3.7 Analysis of expression differences and correlations among six key genes

The heat map illustrates the differences in expression among the six key genes (Figure 6A). There were notable variations in the expression of six genes when comparing the PE and control groups (*p* < 0.001) (Figure 6B). Additionally, these key genes are located on chromosomes 1, 16, 17, 20, 21, and X (Figure 6C). We constructed a correlation heatmap (Figure 6D) and chord diagram (Figure 6E) to depict the correlations within the six key gene expression matrices. Visualizations indicated the strongest positive correlation between *SOD1* and *ALDH4A1* and the most significant negative correlation between *SOD1* and *ABAT*. Friends analysis was conducted to determine functional similarity among the six key genes (Figure 6F), which indicated that *ACSF2* and *ACSS1* showed the most significant functional resemblance to other genes.

**FIGURE 6:**
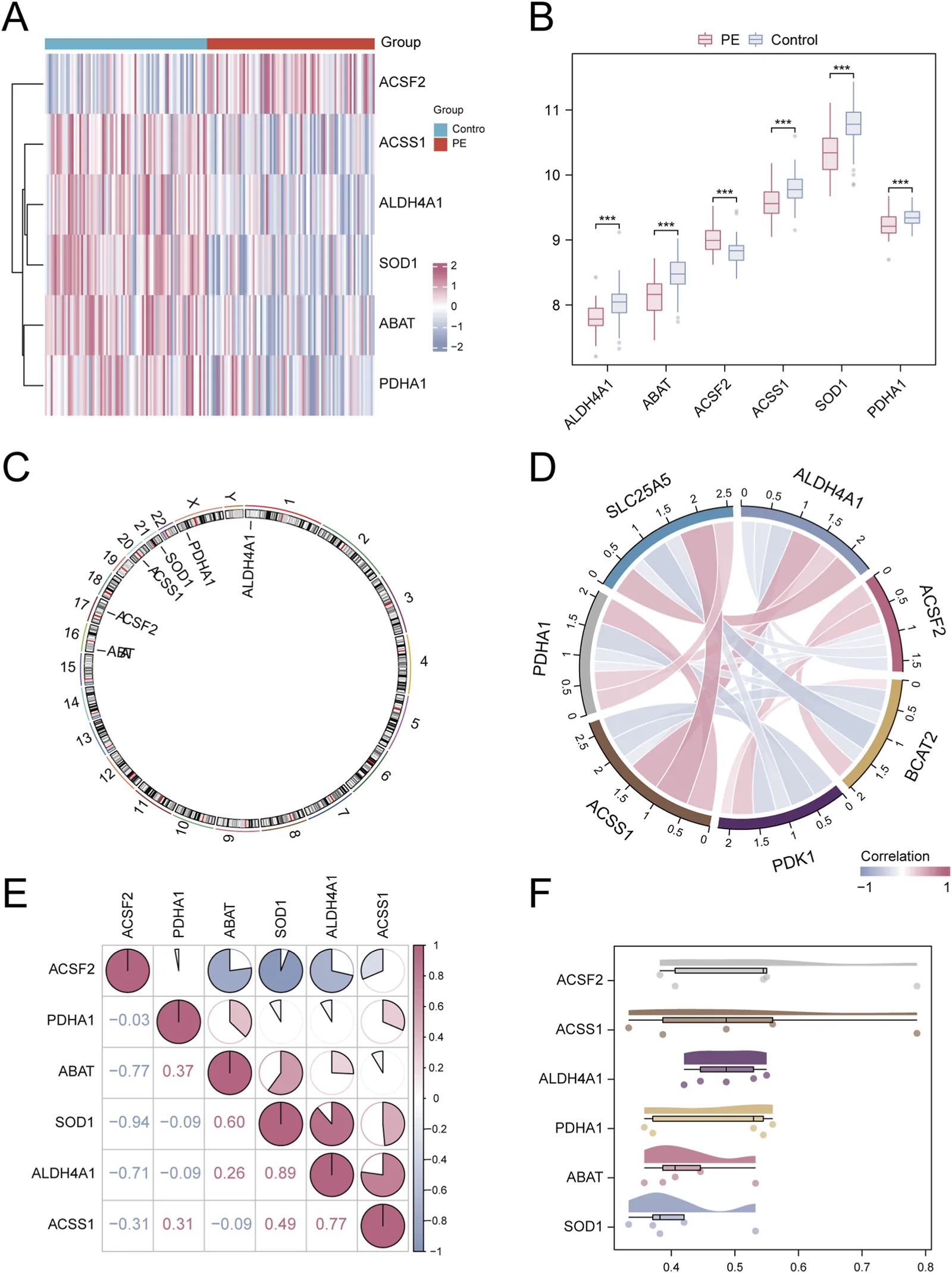
Expression differences and correlation analysis of six key genes. **(A)** Heatmap showcasing the expression levels of six crucial genes across various groups. **(B)** Boxplots depicting the relative expression levels of six key genes in the PE (pink) and control (blue) groups. **(C)** Chromosome localization map of six key genes. **(D,E)** Correlation heatmap and chord diagram illustrating the relationships among six key genes. **(F)** Friends analysis of six key genes. ***p < 0.001.

### 3.8 ROC analysis and construction and interpretation of the predictive model

To enhance the accuracy of predicting the development of PE in patients, we mapped the ROC curves of the six key genes (Figure 7A). Additionally, the ROC curve for the verification set GSE186257 (Figure 7B) showed that *SOD1*, *ACSF2*, *ABAT*, *ACSS1*, and *ALDH4A1* had moderate accuracy (0.7 < AUC <0.9) in the various sample groups. *PDHA1* showed low accuracy (0.5 < AUC <0.7) across the various sample groups. The 10 prediction models established using six key genes all demonstrated good diagnostic efficacy, among which the Random Forest (RF) performed the most excellently (AUC of 0.932, Figure 7C). The calibration curve shows a strong consistency between the predicted and observed PE probabilities (Figure 7D), while the DCA indicates that this model can provide substantial clinical net benefits for clinical decision-making (Figure 7E). SHAP analysis revealed *ABAT* as the most influential feature in the RF model (mean |SHAP| = 0.121), followed by *SOD1* (|SHAP| = 0.083), *ACSF2* (mean |SHAP| = 0.069), *ACSS1* (mean |SHAP| = 0.058), *PDHA1* (mean |SHAP| = 0.033) and *ALDH4A1* (mean |SHAP| = 0.044) (Figure 7F). The expression levels of these genes have different directional effects on PE risk. Elevated *ACSF2* expression correlated with increased PE probability, whereas higher *ABAT*, *SOD1*, *ACSS1*, *PDHA1* and *ALDH4A1* levels exhibited protective effects (Figure 7G). Waterfall plots for representative control (predicted probability = 0.113) and PE (probability = 0.95) cases demonstrated model interpretability, with *ABAT* contributing most substantially to risk prediction (Figures 7H,I).

**FIGURE 7:**
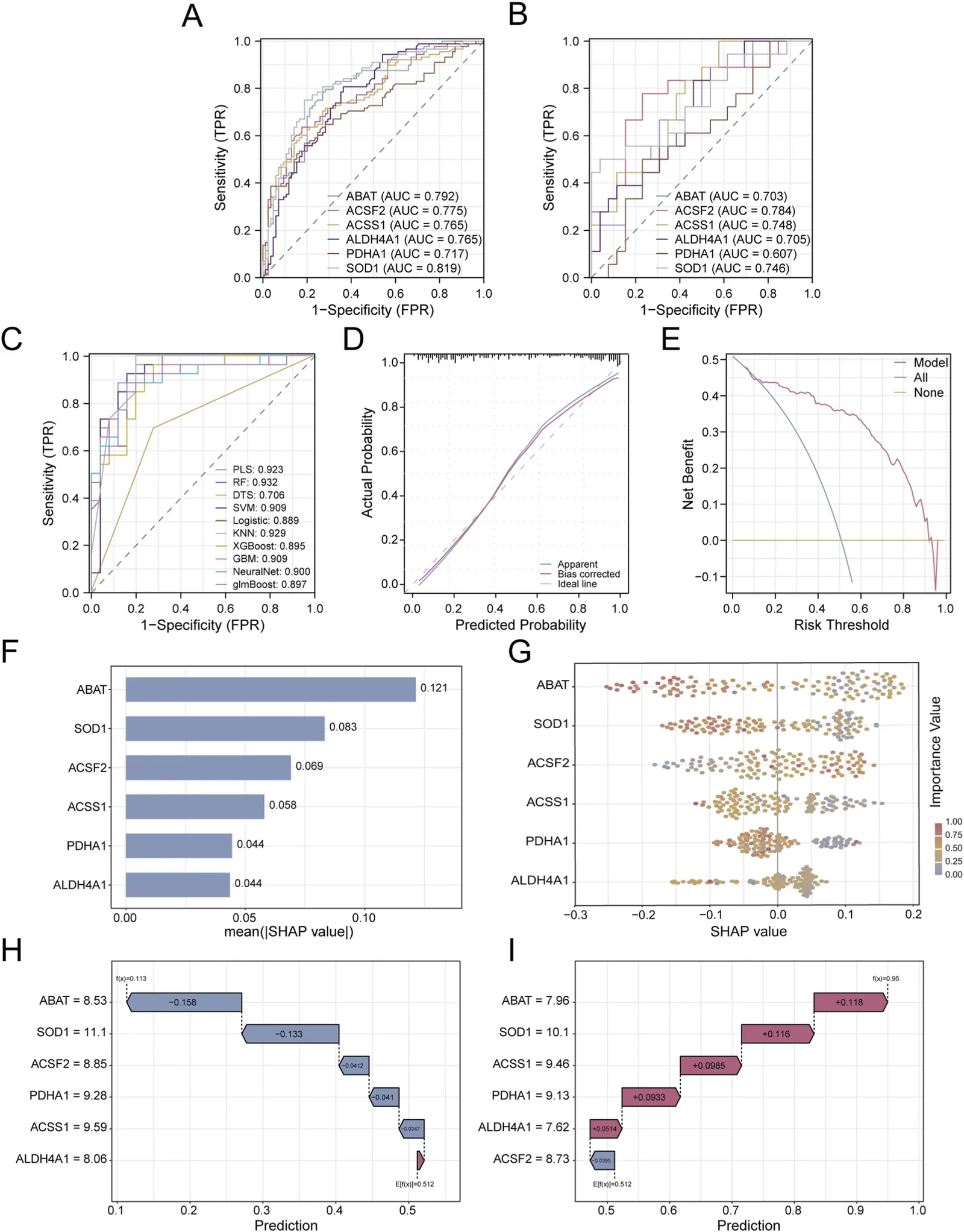
ROC analysis and construction and interpretation of the predictive model. **(A)** ROC curves of the six key genes in the combined dataset. **(B)** ROC curves of the six key genes in the validation dataset GSE186257. **(C)** ROC curves comparing ten machine-learning models. **(D)** Calibration curve of the random forest model. **(E)** Decision curve analysis of the random forest model. **(F)** Feature importance ranked by mean absolute SHAP values. **(G)** Beeswarm plot showing the relationship between gene expression and SHAP values. **(H)** Waterfall plot for a representative control case. **(I)** Waterfall plot for a representative PE case.

### 3.9 Identification and alteration of placental cell subsets in PE

To investigate the microenvironment of placental tissue, we conducted an extensive scRNA-seq analysis on two PE samples and two control samples from dataset GSE173193. Following rigorous quality control, we obtained a total of 27,359 placental cells. We integrated the data from these samples using the Harmony method to correct for batch effects. Subsequently, cell types were annotated based on representative gene markers and visualized using UMAP (Figure 8A). Ultimately, we identified seven distinct cell subsets: granulocytes (marker genes including *S100A8*, *CXCL8*, *MNDA*), trophoblast cells (marker genes including *PAGE4*, *KRT7*), myeloid cells (marker genes including *CD14*, *CD68, CD163*), T/NK cells (marker genes including *CD3D*, *CD3E*, *CD8A*, *KLRB1*, *NCR1*), mast cells (marker genes including *TPSAB1*, *TPSB2*), stromal cells (marker genes including *DKK1*, *IGFBP1*, *PRL*, *CDH5*), and B cells (marker genes including *MS4A1*, *CD19*, *CD79A*). The expression of marker genes across different subsets was depicted using a bubble map (Figure 8B). Furthermore, we characterized these populations by identifying the top five differentially expressed genes for each cell subsets (Figure 8C). We subsequently performed a differential abundance analysis utilizing Milo (Figure 8D). The analysis revealed that, in comparison to the control group, there was an increased proportion of trophoblast cell subsets in the PE group. Conversely, the ratio of myeloid to granulocyte subsets was reduced (Figure 8E). These findings were supported by the proportional analysis of each cell subtype, which confirmed the altered cellular composition associated with the condition (Figure 8F).

**FIGURE 8:**
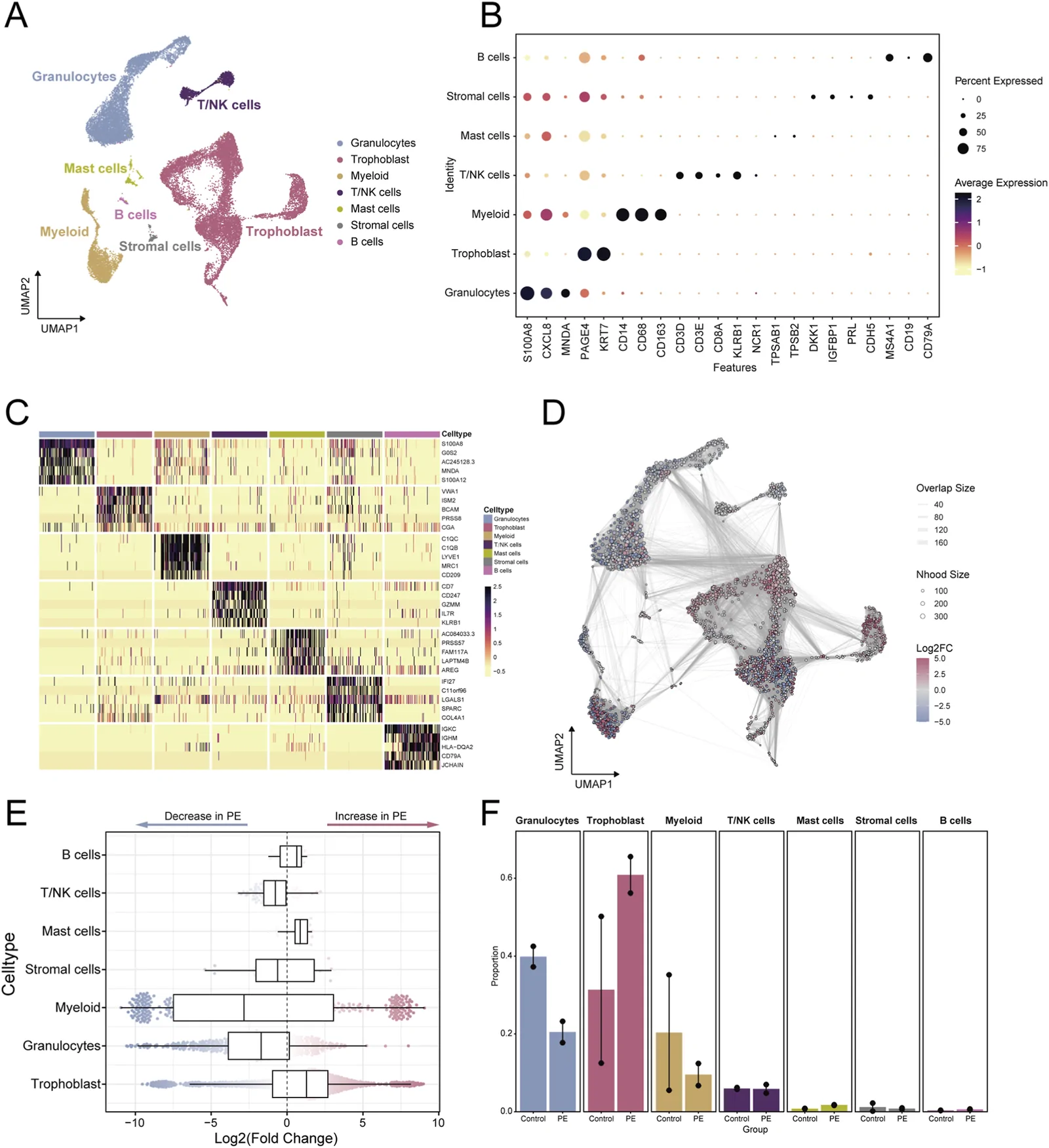
scRNA-seq analysis of placental cells from PE and Control samples. **(A)** UMAP plot illustrating dimensionality reduction and clustering of placental cells, identifying major cell types: granulocytes, trophoblasts cell, myeloid cells, T/NK cells, mast cells, stromal cells, and B cells. **(B)** Dot plot shows marker genes used to distinguish different placental cells subsets. The size of dots represents the percentage of cells expressing marker gene, while the color of dots represents the average expression level of marker gene. The darker the color, the higher the expression level. **(C)** Heatmap presenting the top five differentially expressed genes for each cell subsets. The color scale indicates expression levels, with darker colors signifying higher expression. **(D)** UMAP plot from Milo analysis, illustrating neighborhood overlap and logFC in cell subsets. Nodes represent neighborhoods of cells, edges indicate overlap between neighborhoods, node size corresponds to the overlap size, and the color scale shows the logFC in cell populations, with red indicating an increase and blue a decrease. **(E)** Beeswarm plot from Milo analysis, showing logFC of cell subsets between PE and control groups. **(F)** Bar plot comparing the proportion of each cell subsets between PE and control samples.

### 3.10 AUCell score analysis reveals key mitochondrial activity in cell subsets

The UMAP plot depicts the distribution of cell subsets based on AUCell scores derived from MitoRGs (Figure 9A). The raincloud plot further illustrates the distribution of AUCell scores across different cell subsets (Figure 9B). The results confirmed that the trophoblast cells had the highest AUCell score, thereby indicating that mitochondrial activity was most significant in trophoblast cells. UMAP density plotsdemonstrate the expression levels of key genes (*SOD1*, *ACSF2*, *ABAT*, *ACSS1*, *ALDH4A1*, and *PDHA1*) across different cell subsets. Notably, the genes *SOD1*, *ACSF2*, *ACSS1*, *ALDH4A1*, and *PDHA1* are predominantly expressed in trophoblast cells, whereas *ABAT* is most prominently expressed in myeloid cells (Figures 9C–H). The scatter plot illustrates the expression levels of *SOD1*, *ACSF2*, *ACSS1*, *ALDH4A1*, and *PDHA1* in trophoblast cells (Figure 9I), comparing PE samples with control samples. The waterfall plot demonstrates the differential gene expression in trophoblast cells between PE and control groups (Figure 9J), highlighting the top five upregulated and downregulated genes, thereby offering insights into the most significantly affected genes in PE. The GO and KEGG analysis plotpresents the functional enrichment of differentially expressed genes in trophoblast cells (Figure 9K), revealing enrichment in various BP, CC, MF, and KEGG pathways associated with mitochondrial function.

**FIGURE 9:**
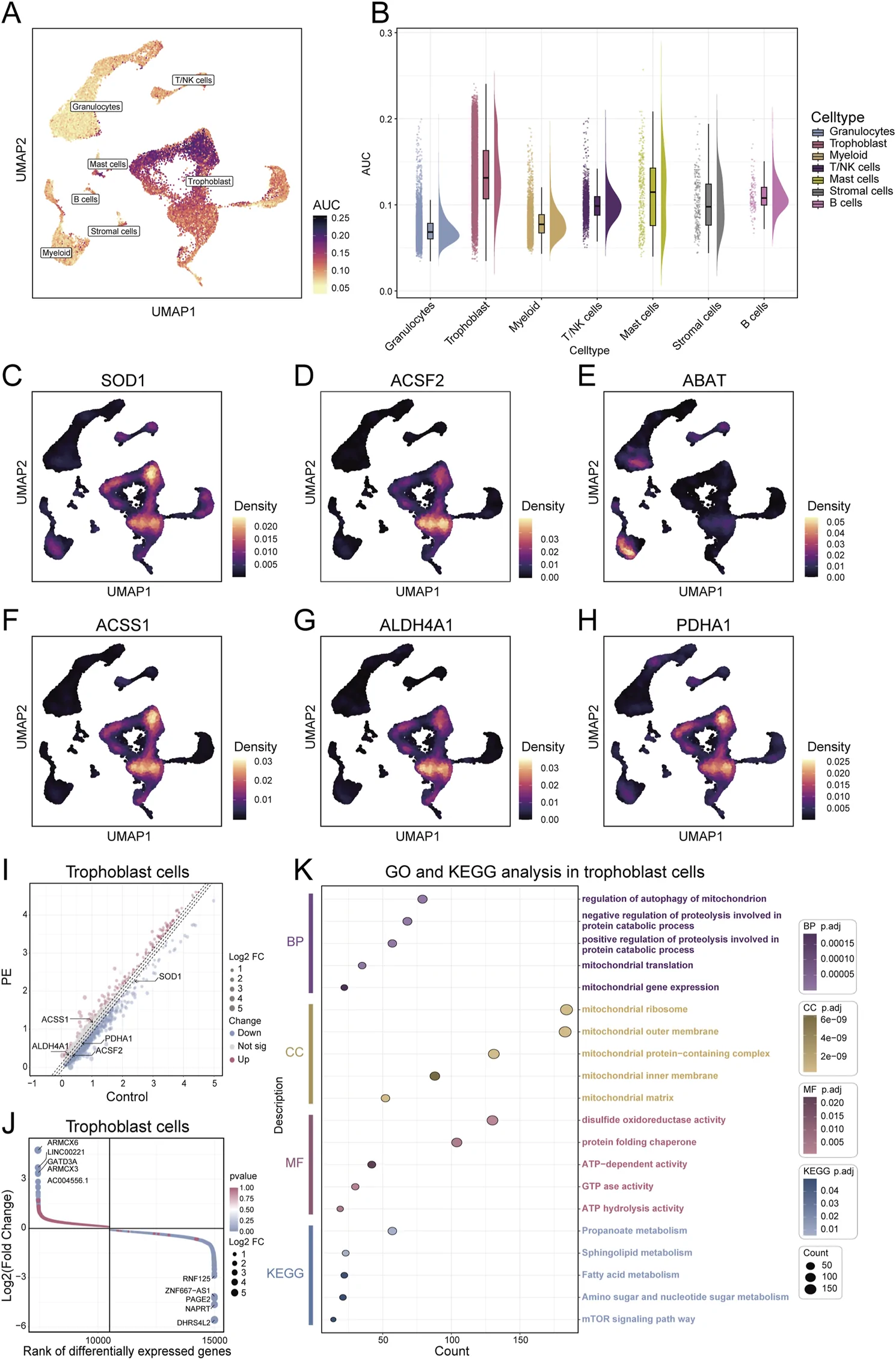
AUCell scoring and key gene analysis in placental cells. **(A)** UMAP plot illustrating the distribution of cell subsets based on AUCell scores derived from MitoRGs. The color gradient represents the AUC scores, with darker shades indicating higher scores. **(B)** Raincloud plot displaying the distribution of AUCell scores across different cell subsets. Each half-violin represents the spread and density of AUC scores for a specific cell type, with the median and interquartile range indicated by the box plot overlay. **(C–H)** UMAP density plots illustrating the expression quantity of key genes across different cell populations. The color gradient indicates the expression level, with light colors representing higher density. **(I)** Scatter plot illustrating the expression of *SOD1*, *ACSF2*, *ACSS1*, *ALDH4A1*, and *PDHA1* between PE and control samples specifically in trophoblast cells. **(J)** Waterfall plot illustrating the differential expression of genes in trophoblast cells between PE and control samples, with red indicating significant upregulation and blue indicating significant downregulation. **(K)** GO and KEGG analysis in trophoblast cells. The color gradient of the dots represents the p. adj for each category, with darker colors denoting higher statistical significance. The size of the dots corresponds to the number of genes associated with each pathway.

### 3.11 Subsets analysis of trophoblast cells reveals altered subset proportions in PE

To investigate the subsets within trophoblast cells, we performed dimensionality reduction clustering and utilized UMAP for visualization (Figure 10A). Consequently, we identified four distinct subsets: progenitor villous cytotrophoblast (VCTp) cells (marker genes including *HMGB2*, *CCNB2*, *CDK1*, *TOP2A*, *AURKA*, *AURKB*, *CDKN3*, and *PLK1*), villous trophoblast (VCT) cells (marker genes including *SLC27A2*, *SLC52A1*, and *FOLR1*), syncytiotrophoblast (SCT) cells (marker genes including *CGA*, *GDF15*, *SDC1*, *PGF*, and *HSD17B1*), and extravillous trophoblast (EVT) cells (marker genes including *HLA-G*,*PAPPA2*), Furthermore, the expression of marker genes across different subsets was illustrated using a bubble diagram (Figure 10B). Concurrently, we depicted the distribution of four subsets within the overall sample (Figure 10C). The accumulation histogram revealed an increased proportion of VCTp cells and EVT cells in the PE group compared to the control group, whereas the proportion of VCT cells decreased (Figure 10D). The subcells enrichment map demonstrated the functional enrichment of differentially expressed genes across various trophoblast cell subsets. The findings suggest that numerous KEGG pathways associated with mitochondrial function are enriched in the samples (Figure 10E).

**FIGURE 10:**
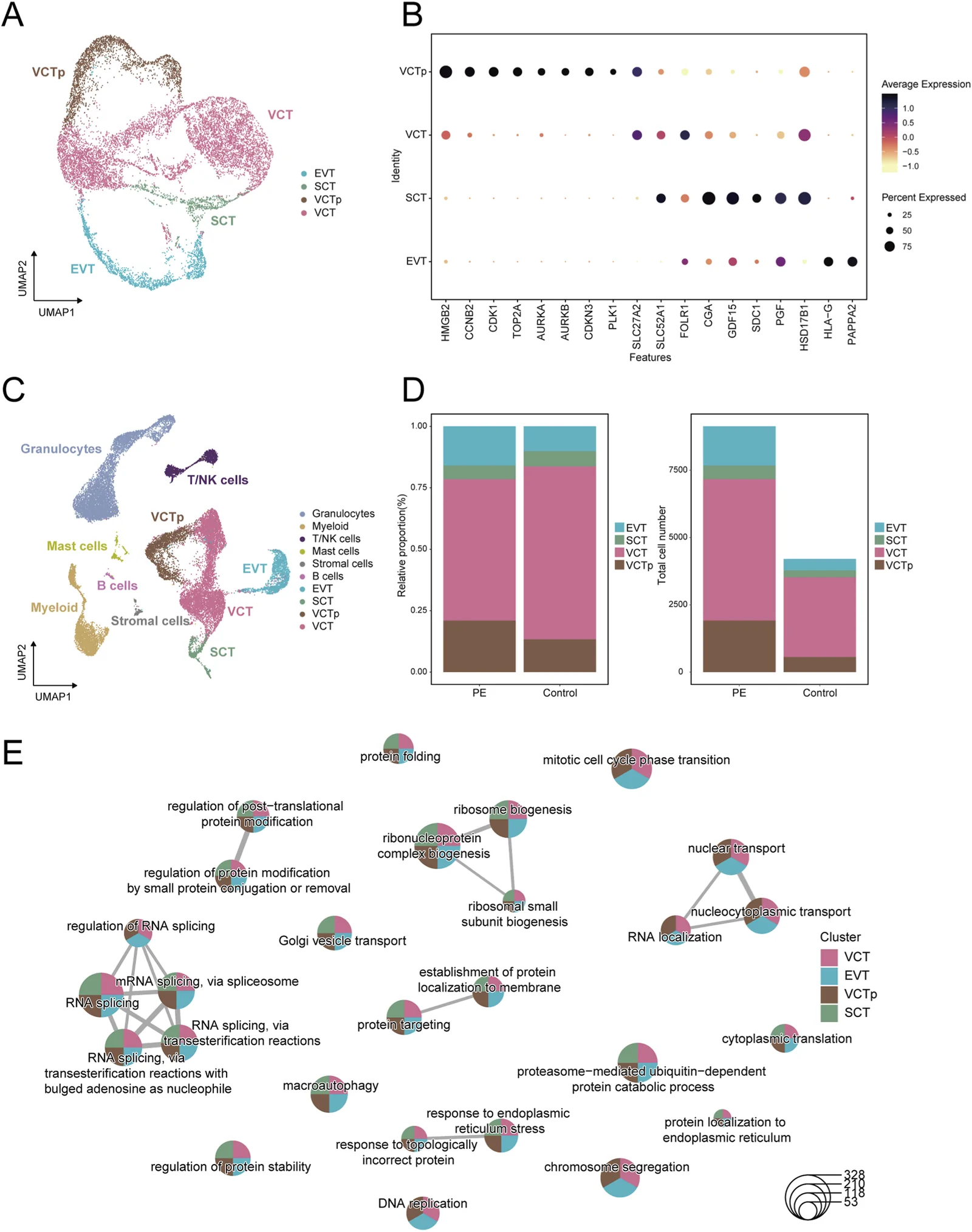
Enrichment analysis of further groups and subsets of trophoblast cells. **(A)** UMAP plot illustrating dimensionality reduction and clustering of trophoblast cells, identifying major cell types: VCTp cells, VCT cells, SCT cells, and EVT cells. **(B)** Dot plot shows marker genes used to distinguish different trophoblast cells subsets. The size of dots represents the percentage of cells expressing marker gene, while the color of dots represents the average expression level of marker gene. The darker the color, the higher the expression level. **(C)** UMAP shows the distribution of trophoblast subsets in placental cells. **(D)** Bar plots showing the ratio of each trophoblast cell subsets to all trophoblast cells in the PE and control groups (left) and the number of cells per subsets (right). **(E)** Subsets enrichment map displaying the proportional KEGG pathways analysis for all trophoblast cell substs. The size of each node indicates the number of genes within each KEGG category.

### 3.12 Cell trajectory analysis reveals differentiation pathways in trophoblast cell subsets

In the analysis of cell trajectories, we initially assessed the differentiation status of various cell subsets utilizing CytoTRACE2 scores (Supplementary Figure S2A). The scores revealed that distinct cell types possessed varying developmental potentials, with VCTp cells exhibiting the highest score, indicative of the lowest differentiation status among the cell subsets. Subsequently, we employed Monocle 3 to construct a developmental trajectory map, illustrating the pseudo-temporal progression of the cells (Supplementary Figure S2B). By integrating the CytoTRACE2 score, we determined VCTp cells as the starting point of the cell development trajectory. VCTp cells initially differentiated into VCT cells, with some VCT cells further differentiating into SCT cells and EVT cells (Supplementary Figure S2C). Finally, we investigated cell differentiation trajectories in three-dimensional space using 3D diffusion maps, which offer a more comprehensive perspective on cell spatial distribution and differentiation pathways by incorporating the relative order of CytoTRACE2 scores (Supplementary Figure S2D). The diffusion components (DC1, DC2, and DC3) delineate trajectories of cell subpopulations that align with developmental pathways inferred from Monocle 3 (Supplementary Figure S2E).

### 3.13 Cell-cell interaction analysis reveals communication between cell subsets in PE

In the analysis of cell-cell interactions, the CellChat R package was employed to examine the communication dynamics among various cell subsets within the PE group. Initially, interaction frequency and strength were visualized using circle plots, which identified VCT cells as central to the communication network, demonstrating both high interaction frequency and strength (Supplementary Figures S3A,B). We further investigated the Vascular endothelial growth factor (VEGF) signaling pathway (Supplementary Figure S3C) and emphasized the most significant ligand-receptor pair within it: *VEGFA*-*VEGFR1* (Supplementary Figure S3D). The results indicated that in the VEGF signaling pathway, EVT cells contributed the highest in the incoming interaction strength, while SCT cells contributed the highest in the outgoing interaction strength (Supplementary Figure S3E). Meanwhile, we also studied the Macrophage migration inhibitory factor (MIF) signaling pathways (Supplementary Figure S3F) and emphasized the most significant ligand-receptor pair within it: *MIF*-(*CD74*+*CXCR4*) (Supplementary Figure S3G). The results indicated that in the MIF signaling pathway, B cells contributed the highest in the incoming interaction strength, while EVT cells and VCTp cells contributed the highest in the outgoing interaction strength (Supplementary Figure S3H). Finally, dot plots illustrated the outgoing and incoming communication patterns of secreting and target cells, respectively, offering a comprehensive view of the signaling dynamics within the PE group (Supplementary Figure S3I,J).

### 3.14 Validation of key genes in PE

We performed RT-qPCR to examine the expression levels of six critical genes in PE. The analysis included ten patients with PE and ten matched placental samples based on gestational age. The primer sequences are provided in Table 3. RT-qPCR analysis revealed significantly reduced expression levels of *ABAT*, *PDHA1* and *SOD1* in placental samples from PE patients compared to the control group (Figures 11A–F).

**TABLE 3:** Primer sequences for RT-qPCR.

Genes	Forward primer (5′-3′)	Reverse primer (5′-3′)
*SOD1*	GGTGGGCCAAAGGATGAAGAG	CCACAAGCCAAACGACTTCC
*ACSF2*	CCAGGCTATGGAACTGGAGTA	CTGGGTCTTGAATTGCTTGGG
*ABAT*	TGGGTTGCTTAGCGACCAC	TCCAGAGGGTATTTCAGCCGT
*ACSS1*	GCCGACATCGGTTGGATTACA	GTGCTCTCAAAAAGGACGCTG
*ALDH4A1*	CAGGGTAAGACCGTGATCCAA	CCAGCTCCACCGCATACTTG
*PDHA1*	TGGTAGCATCCCGTAATTTTGC	ATTCGGCGTACAGTCTGCATC
*GAPDH*	AGTATGATTCCACCCACGGC	GATGGCCTTCCCGTTGATGA

**FIGURE 11:**
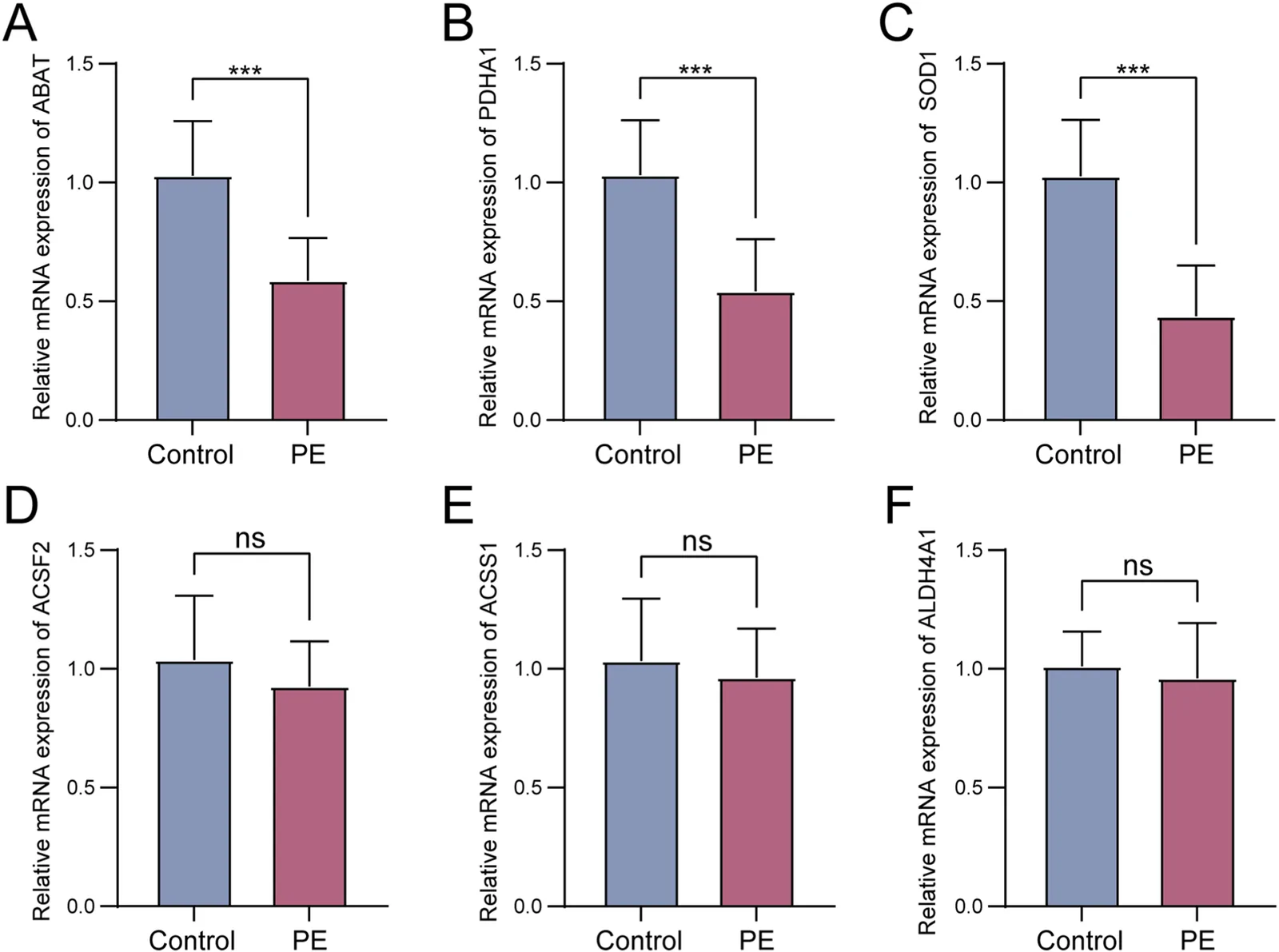
Expression of key genes in placental samples from control versus PE groups. The expression levels of key genes *ABAT* **(A)**, *PDHA1* **(B)**, *SOD1* **(C)**, *ACSF2* **(D)**, *ACSS1* **(E)**, and *ALDH4A1* **(F)**, in the control and PE groups are illustrated through expression bars. ***p < 0.001.

## 4 Discussion

The peripartum management of PE constitutes a major clinical challenge in obstetrics due to its unpredictable trajectory, with potential rapid progression to life-threatening complications including eclamptic seizures, HELLP syndrome (hemolysis, elevated liver enzymes, low platelets), and multiorgan dysfunction (Author Anonymous, 2020). Despite decades of research, the precise etiopathogenesis remains enigmatic, hampering the development of targeted therapies. Current diagnostic criteria—reliant on *de novo* hypertension (≥140/90 mmHg) and proteinuria (≥300 mg/24 h)—lack predictive value for disease progression (Brown et al., 2018). Therapeutic strategies remain predominantly reactive, focusing on blood pressure control, eclampsia prophylaxis, and expedited delivery—the only definitive intervention but one that carries risks of iatrogenic prematurity (Dimitriadis et al., 2023). This diagnostic and therapeutic impasse underscores the urgent need to elucidate the molecular underpinnings of PE pathogenesis.

Mitochondria are pivotal in PE pathogenesis through a triad of interconnected mechanisms: energy metabolism collapse, oxidative stress amplification, and epigenetic dysregulation. As the cellular powerhouses, mitochondria drive ATP production via oxidative phosphorylation (OXPHOS) and the TCA cycle. In PE, impaired mitochondrial respiratory chain complexes lead to ATP deficiency, forcing placental cells into glycolytic dependency—a metabolic shift that acidifies the microenvironment and disrupts trophoblast invasion (Aye et al., 2022; Holland et al., 2017). Concurrently, excessive ROS generation overwhelms antioxidant defenses, triggering lipid peroxidation, mtDNA damage, and caspase-mediated apoptosis (Hu and Zhang, 2022). These processes are further exacerbated by mitochondrial dysfunction-induced epigenetic alterations: critical metabolites required for histone modifications and DNA methylation become depleted, potentially silencing genes essential for placental development and immune tolerance (Matilainen et al., 2017). This triad—metabolic paralysis, oxidative toxicity, and epigenetic rewiring—orchestrates PE’s pathological cascade, where MitoRGs play a central role in both its occurrence and progression. However, the fundamental biological mechanisms of MitoRGs remain poorly understood; further research into these genes could not only uncover novel biomarkers enabling pre-symptomatic identification of high-risk pregnancies but also facilitate the development of pathogenesis-targeted therapies that address core disease mechanisms rather than merely alleviating clinical manifestations. Such innovations promise to revolutionize PE care by shifting from reactive management to precision prevention, optimizing maternal-fetal outcomes while curtailing prematurity-related morbidity.

This study systematically deciphered the tripartite regulatory axis of mitochondrial dysfunction in PE through integrative multi-omics, machine learning, and experimental validation, elucidating how mitochondrial-related genes (MitoRGs) drive PE progression via energy metabolism collapse, oxidative stress amplification, and epigenetic dysregulation. We identified six core genes—*ABAT*, *PDHA1*, *SOD1*, *ACSF2*, *ACSS1*, and *ALDH4A1*—with aberrant expression in PE placentas. RT-qPCR validation confirmed *ABAT*, *PDHA1*, and *SOD1* as robust diagnostic markers and therapeutic targets. Functionally, these genes orchestrate three interlinked pathological axes in PE: (1) energy metabolism collapse via TCA cycle disruption and fatty acid oxidation impairment, (2) ROS homeostasis breakdown through compromised antioxidant defense, and (3) metabolite-driven epigenetic modulation mediated by mitochondrial substrate availability (e.g., acetyl-CoA for histone acetylation). Together, these findings position mitochondrial dysfunction as a central hub integrating metabolic failure, redox imbalance, and epigenetic reprogramming in PE pathogenesis, offering novel avenues for biomarker discovery and mechanism-targeted therapies.

Mitochondrial energy metabolism disorder is the core driving force of placental failure in PE. Mitochondria generate ATP through the tricarboxylic acid cycle (TCA cycle) and oxidative phosphorylation (OXPHOS). The significant downregulation of key enzymes *PDHA1* and *ACSS1* in the TCA cycle in PE placentas directly leads to the obstruction of pyruvate conversion to acetyl-CoA and a reduction in ATP synthesis capacity (Andrejeva and Rathmell, 2017)。*PDHA1* (*alpha 1 subunit of the pyruvate dehydrogenase E1*), as the rate-limiting subunit of the pyruvate dehydrogenase complex, its downregulation forces cells to rely on glycolysis for energy supply, generating large amounts of lactic acid (Sun et al., 2019), causing acidification of the placental microenvironment, and inhibiting trophoblast cell invasion and vascular remodeling. Single-cell sequencing shows that *PDHA1* is specifically lowly expressed in trophoblast cells, while the proportion of VCTp cells increases and that of VCT cells decreases in the PE group, suggesting that it may attempt to alleviate the energy crisis through compensatory proliferation. This metabolic reprogramming conforms to the “Warburg effect”, that is, cells shift to inefficient glycolysis under energy stress, which can maintain survival temporarily but exacerbates placental dysfunction (Fan et al., 2014). In addition, *ACSS1* (*Acyl-CoA synthetase short chain family member 1*) catalyzes the conversion of short-chain fatty acids and CoA to acetyl-CoA (a key intermediate of the TCA cycle), and its expression is significantly downregulated in PE, resulting in reduced acetyl-CoA production. This not only inhibits mitochondrial energy metabolism but may also interfere with epigenetic regulation by limiting the supply of histone acetylation substrates (Gao et al., 2016), thereby participating in the long-term pathological memory of PE.

Mitochondrial oxidative stress is a key amplifier of placental damage in PE. Mitochondria are the main source of reactive oxygen species (ROS), and the significant downregulation of the antioxidant enzyme *SOD1* leads to the collapse of ROS clearance capacity. *SOD1* (*Superoxide dismutase 1*), as a superoxide dismutase, its functional deficiency causes the accumulation of superoxide anions (O_2_^−^). ROS can not only directly induce mitochondrial membrane lipid peroxidation and mtDNA mutations, activate the Caspase-9-dependent apoptotic pathway, but also promote the release of pro-inflammatory factors (such as TNF-α, IL-6) through the NF-κB pathway, activating the systemic inflammatory response in the mother (Burton and Jauniaux, 2011). Moreover, ROS can enhance sFlt-1 expression by modifying HIF-1α, inhibit the VEGF signaling pathway, and cause vascular endothelial dysfunction (Eddy et al., 2023). This “pro-inflammatory-anti-angiogenic” interaction network continuously amplifies oxidative damage through a positive feedback loop, ultimately forming an irreversible pathological microenvironment, which is highly consistent with the clinical manifestations of PE patients, such as hypertension and proteinuria. Notably, oxidative stress and energy metabolism disorder form a vicious cycle: the accumulation of pyruvate caused by the downregulation of *PDHA1* enters the cytoplasm through mitochondrial retrograde transporters, is converted to lactic acid, and is accompanied by NAD + consumption, further weakening the cell’s antioxidant capacity and exacerbating oxidative damage.

The abnormal expression of *ABAT* (*4-aminobutyrate aminotransferase*) reveals the imbalance of amino acid metabolism and neuroimmune regulation in PE. *ABAT* catalyzes the conversion of GABA to succinic semialdehyde, connecting neurotransmitter metabolism with the TCA cycle. This study found that the expression of *ABAT* in PE placental tissues was significantly decreased. The accumulation of GABA caused by the absence of *ABAT* not only enters the matrix through mitochondrial retrograde transporters, inhibits the activity of α-ketoglutarate dehydrogenase, weakens TCA cycle energy metabolism, and exacerbates placental energy crisis (Fujii and Mellon, 2001), but also inhibits the differentiation of regulatory T cells (Treg) by activating GABAA receptors and promotes the polarization of Th17 cells (Han et al., 2024), which is consistent with the clinical observation of Th17/Treg immune imbalance in the peripheral blood of PE patients. The RT-qPCR verification results of *ABAT* provide direct evidence for its role as a marker of metabolic disorder in PE, suggesting that it may become a potential indicator for assessing the risk of PE complications.

The abnormal expression of *ACSF2* and *ALDH4A1* further reveals the extensive dysregulation of lipid and amino acid metabolism in PE. *ACSF2* (*Acyl-CoA synthetase family member 2*) shows expression heterogeneity in PE: although its expression is elevated in the overall placental tissue, single-cell sequencing reveals a specific downregulation of *ACSF2* in trophoblast cells, leading to impaired fatty acid metabolism, lipid accumulation, and the accumulation of toxic products within this cell subpopulation (Wang et al., 2019). This cell type-specific metabolic imbalance suggests that although the placenta attempts to compensate for the energy crisis through global activation of fatty acid metabolism, the local mitochondrial lipotoxic damage in trophoblast cells eventually overwhelms the compensatory mechanism, exacerbating placental dysfunction. *ALDH4A1* (*Aldehyde dehydrogenase 4 family member A1*), which is involved in proline-glutamate metabolism, when downregulated, may disrupt the cellular redox balance, causing proline accumulation, activating the endoplasmic reticulum stress pathway, and promoting the unfolded protein response (UPR) to increase the expression of apoptosis-related genes, thereby forming a “metabolism-endoplasmic reticulum stress-apoptosis” cascade reaction (Vasiliou and Pappa, 2000).

This study constructed a PE prediction model based on mitochondrial-related genes (MitoRGs) through multiple machine learning algorithms and utilized the SHAP explainability framework to reveal the contributions of key genes, providing a multi-dimensional perspective for uncovering the molecular mechanisms of PE. This modeling strategy not only overcame the insensitivity of traditional single-gene markers but also revealed the complex pathological network of PE through the perspective of multi-gene synergy. The random forest model performed the best among the ten algorithms and significantly outperformed traditional clinical indicators (such as the sFlt-1/PlGF ratio, etc.). Its calibration curve showed a high consistency between predicted probabilities and actual risks, and the decision curve analysis further confirmed that this model could provide significant net benefits in clinical decision-making. SHAP analysis further quantified the contribution of each gene to the model prediction, identifying *ABAT* and *SOD1* as key drivers. Their negative SHAP values were highly consistent with experimental verification results, which was highly consistent with their protective functions in energy metabolism and oxidative stress, confirming that energy metabolism and oxidative stress play a core role in mitochondrial disorders. The low expression of *ABAT* exacerbated TCA cycle blockage through GABA metabolic disorder and mediated Th17/Treg immune imbalance through GABAA receptor, which was manifested as a strong contribution to high-risk prediction in the SHAP waterfall plot. The low expression of *SOD1* led to a burst accumulation of mitochondrial ROS by weakening the superoxide scavenging ability, directly activating the NF-κB inflammatory pathway and enhancing HIF-1α/sFlt-1 axis-mediated vascular endothelial injury. Single-cell data revealed its specific downregulation in trophoblast cells, further amplifying the local effect of oxidative stress. This dose-dependent risk pattern was manifested as a high contribution of *SOD1* to high-risk prediction in model interpretation. Notably, *ACSF2*, as a risk factor, showed bidirectional regulatory characteristics in bulk RNA-seq and scRNA-seq: although its overall placental expression increased, single-cell resolution revealed its specific downregulation in trophoblast subpopulations, leading to local lipotoxic damage. The SHAP explanation diagram of a representative PE sample confirmed this, with the expression level of *ACSF2* showing a negative contribution to the model’s prediction, suggesting that the local explainability of machine learning models can more accurately map the pathological complexity of gene expression levels.

From a clinical translation perspective, this model, by integrating multi-omics data with machine learning algorithms, has broken through the limitations of traditional diagnostic markers (such as proteinuria and hypertension), achieving stratification of PE risk. The SHAP-driven ranking of feature importance not only provides a basis for individualized risk assessment but also guides the prioritization of potential therapeutic targets. However, because placental tissues were predominantly collected at delivery, the current findings should be considered a discovery-stage placental molecular signature rather than a clinically validated early diagnostic model. Prospective studies using maternal blood or placenta-derived extracellular vesicles collected before clinical onset are required to determine temporal changes, specificity, and incremental predictive value beyond established indicators such as the sFlt-1/PlGF ratio.

While this study provides novel insights into mitochondrial dysfunction in PE, several limitations warrant consideration. First, the reliance on bulk RNA sequencing from public databases introduces inherent heterogeneity in sample collection protocols and clinical classifications across cohorts. Although rigorous batch correction was applied, residual confounding from unmeasured variables may persist. Moreover, the retrospective and cross-sectional nature of the placental datasets precluded assessment of temporal relationships. Consequently, we cannot determine whether the six genes contribute to PE development, precede its clinical onset, or represent downstream consequences of established disease. The reported AUC values quantify discrimination within the analyzed placental datasets and do not demonstrate early predictive performance. Prospective longitudinal studies using clinically accessible maternal samples collected before PE onset are necessary before these genes can be considered early biomarkers. Second, the scRNA-seq validation cohort included only four samples (2 PE, 2 controls), limiting statistical power to detect subtle cellular heterogeneity. Third, our analysis focused exclusively on placental tissue, whereas mitochondrial dysfunction in PE likely involves systemic crosstalk between maternal endothelium, immune cells, and extraplacental tissues—a dimension not captured here. Fourth, although the machine learning model performed well in the independent validation set, its clinical transformation still needs to be further verified through a prospective multi-center cohort. Finally, functional validation was restricted to mRNA quantification via RT-qPCR; future studies should incorporate protein-level assays and mechanistic experiments using trophoblast cell lines or organoids to validate gene functions. Future research should prioritize three directions: (1) Multi-omics integration–Combining proteomic and metabolomic profiling with spatial transcriptomics could elucidate how mitochondrial gene dysregulation alters placental nutrient transport and hormonal signaling. (2) Mechanistic studies–CRISPR-based modulation of key genes in trophoblast models could clarify their roles in redox balance and immune regulation. Animal models may test whether mitochondrial-targeted antioxidants mitigate disease progression. (3) Clinical translation–non-invasive biomarkers reflecting placental mitochondrial stress should be explored for early risk stratification.

## 5 Conclusion

This study elucidates the pivotal role of mitochondrial dysfunction in PE pathogenesis through an integrative approach combining multi-omics bioinformatics, machine learning, and experimental validation. We identified six MitoRDEGs—*ABAT*, *PDHA1*, *SOD1*, *ACSF2*, *ACSS1*, and *ALDH4A1*—as PE-associated placental mitochondrial genes. These genes were associated with three pathological processes: (1) energy metabolism disruption involving of the TCA cycle and fatty acid oxidation, (2) oxidative stress amplification due to impaired ROS scavenging, and (3) immune and epigenetic dysregulation related to metabolite imbalances. RT-qPCR validation confirmed significant downregulation of *ABAT*, *PDHA1*, and *SOD1* in PE placentas, supporting their association with PE. Single-cell RNA sequencing revealed cell-type-specific dysregulation associated with altered cellular composition. Machine-learning models, particularly the Random Forest algorithm, demonstrated good discrimination between PE and control placental samples in the analyzed retrospective datasets, with SHAP analysis highlighting *ABAT* and *SOD1* as important contributors to model prediction. These findings support an association between placental mitochondrial dysfunction and metabolic, oxidative, and epigenetic alterations in PE. However, because the placental samples were predominantly collected at delivery, the temporal roles of these genes and their potential as early biomarkers require validation in prospective longitudinal cohorts.

## Data Availability

The publicly available datasets analyzed in this study are available in the NCBI Gene Expression Omnibus (GEO) repository under accession numbers GSE24129, GSE75010, GSE186257, and GSE173193. Further inquiries may be directed to the corresponding author.
