# Supplementary material for: Integrated bioinformatics and single-cell transcriptomics identify mitochondrial dysfunction-driven biomarkers for preeclampsia diagnosis
Source: Front Mol Biosci. 2026 Sep 23;13:1922190. doi: 10.3389/fmolb.2026.1922190 (PMC13642145; doi:10.3389/fmolb.2026.1922190)
Supplement: Supplementary file 1 [file Table1.docx]

**Figure S1 Combined dataset for data cleansing.** (A) combined dataset distribution boxplots before normalization. (B) combined dataset distribution boxplots after normalization. (C) combined dataset PCA plot before normalization. (D) combined dataset PCA plot after normalization.

**Figure S2 Developmental trajectory analysis of trophoblast subsets.** (A)Boxplot illustrating CytoTRACE2 scores among VCTp cells, VCT cells, SCT cells, and EVT cells. (A)Boxplot illustrating CytoTRACE2 scores among VCTp cells, VCT cells, SCT cells, and EVT cells. (B)Monocle3 UMAP plot showing pseudotime trajectory starting from VCTp cells. Dark colors indicate that the cell is in an early stage of the developmental process, while light colors indicate that the cell is further along the trajectory toward a more differentiated state. (C) Monocle3 UMAP plot with cell subsets shows the location of VCTp cells, VCT cells, SCT cells, and EVT cells on the pseudotime trajectory. (D)Developmental trajectory analysis using 3D Diffusion Map combined with CytoTRACE2 relative order to assess the developmental trajectory of trophoblast cells. The higher the score, the closer the cell is to an undifferentiated state. (E)3D diffusion map with cell subsets showed the localization of VCTp cells, VCT cells, SCT cells, and EVT cells on the developmental trajectory.

**Figure S3 Analysis of cell subsets interactions and signaling pathways in the PE group.** (A) The number of interactions between cell subpopulations in the PE group. The color of the line indicates the sender of the signal, and the thickness of the connecting lines indicates the frequency of interactions between them. (B) The interaction weights/strengths between cell subpopulations in the PE group. The color of the line indicates the sender of the signal, and the thickness of the lines reflects the strength of their interactions. (C) Circle plot of the inferred VEGF signaling pathway network in the PE group. The color of the line indicates the sender of the signal, and the thickness of the lines reflects the strength of their interactions. (D) Chord plot of the most significant ligand-receptor pair within the VEGF signaling pathway: VEGFA - VEGFR1. The color of the line indicates the sender of the signal, and the width of the lines indicates the direction and intensity of the interaction between cell subpopulations. (E) Scatter plot showing the contribution of different cell subsets of VEGF signaling pathways to efferent and afferent networks. The size of the dots indicates the count of cells involved. (F) Circle plot of the inferred MIF signaling pathway network in the PE group. (G) Chord diagram of the most significant ligand-receptor pair within the MIF signaling pathway: MIF - (CD74+CXCR4). (H) Scatter plot showing the contribution of different cell subsets of MIF signaling pathways to efferent and afferent networks. (I) Dot plot showing outgoing communication patterns of secreting cells. The size of the dots reflects the level of contribution of that cell subpopulation to the pathway. (J) Dot plot showing incoming communication patterns of target cells.
